# On the synthesis and structure of reactive halonium ions†

**DOI:** 10.1039/d5sc03756e

**Published:** 2025-06-19

**Authors:** Lukas Fischer, Michael H. Lee, Anja Wiesner, Carsten Müller, Sebastian Riedel

**Affiliations:** a Fachbereich Biologie, Chemie, Pharmazie, Institut für Chemie und Biochemie – Anorganische Chemie, Freie Universität Berlin Fabeckstraße 34/36 14195 Berlin Germany s.riedel@fu-berlin.de

## Abstract

Fluorinated diethyl and dipropyl halonium salts, [Br(CH_2_CF_3_)_2_][WCA] 1Br, [I(CH_2_CHF_2_)_2_][WCA] 2I and [X(CH_2_CH_2_CF_3_)_2_][WCA] (X = Br 5Br, I 5I; WCA = [Sb(OTeF_5_)_6_]^−^), were synthesized *via* fluoroalkylation or oxidation of bromofluoro and iodofluoro alkanes. The molecular structures of 1Br and 2I, as well as a second different solid-state structure of the previously reported chloronium salt 1Cl, were determined by single-crystal X-ray diffraction. Additionally, the oxidation of 2-chloro-1,1,1-trifluoropropane CHCl(CH_3_)(CF_3_) led to the formation of a highly reactive compound that activates isobutane to form the *tert*-butyl cation [C_4_H_9_]^+^. Although the product could not be directly observed due to its thermal instability and low solubility, quantum-chemical calculations suggest the formation of an asymmetric chloronium ion with trifluoro *n*-propyl and isopropyl side chains.

## Abstract 2

Fluorinated diethyl and dipropyl halonium salts were synthesized *via* fluoroalkylation of bromofluoro and iodofluoro alkanes. Oxidation of CHCl(CH_3_)(CF_3_) yielded a highly reactive asymmetric chloronium species, capable of isobutane activation.

## Graphical abstract

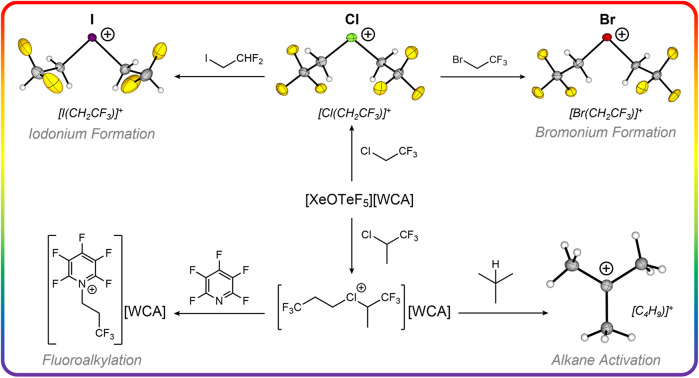

## Introduction

Numerous stable diaryl and aryl-alkyl halonium ions have been reported in the literature. For example, iodonium salts derived from these compound classes are widely employed as efficient aryl or alkyl transfer reagents for electron-rich nucleophiles.^1^ However, the chemistry of diaryl and aryl-alkyl bromonium salts remains largely underexplored, whereas recent studies by Miyamoto and Uchiyama have highlighted the use of diaryl chloronium salts as potent arylation agents.^2^ A variety of stable diaryl halonium ions have been reported, but compounds containing dialkyl halonium ions are still rare. Since the early studies by Olah in this field,^3–6^ only a few reports on the synthesis and application of these reactive ions can be found, with the noteworthy report on the first [C–F–C]^+^ fluoronium ion in solution and the solid state.^7^ This ion exhibits a sophisticated cage-like structure and is obtained by fluoride abstraction from the difluorinated double-norbornyl type precursor. The preparation of simpler dimethyl halonium ions [X(CH_3_)_2_]^+^ (X = Cl, Br, I) can be achieved using multiple pathways such as methylation,^3–5,8^ protonation,^6,9^ halide abstraction^3–5^ or halide oxidation^10,11^ of the corresponding halomethane CH_3_X. These dimethyl halonium ions are powerful electrophilic methylation reagents with increasing reactivity in the order [I(CH_3_)_2_]^+^ < [Br(CH_3_)_2_]^+^ < [Cl(CH_3_)_2_]^+^.^6^ Recently, our group reported on the synthesis of the fluorinated dialkyl halonium salts [X(CH_2_CF_3_)_2_][E(OTeF_5_)_*n*_] (X = Cl, I; E = Al, *n* = 4; E = Sb, *n* = 6).^10^ While the iodonium salts are able to alkylate weak nucleophiles like 2,6-difluoropyridine, the chloronium salts showed exceptionally high reactivity, not only acting as fluoroalkyl transfer reagents but also in the activation of alkanes like *n*-pentane or *n*-butane, yielding branched carbocations.

To follow up on this chemistry, we were interested in the synthesis of more fluorinated dialkyl halonium salts with various alkyl substituents. The hitherto unknown fluorinated dialkyl bromonium salts are especially interesting, with an expected higher alkylation power than iodonium salts combined with a higher stability and more controlled reactivity than chloronium salts.

## Results and discussion

**Scheme 1:**
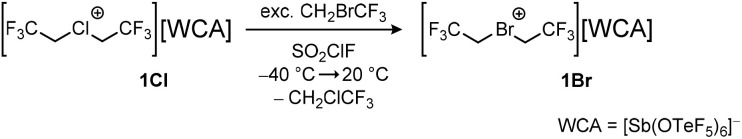
Synthesis of the bromonium salt 1Br.

The bromonium salt [Br(CH_2_CF_3_)_2_][Sb(OTeF_5_)_6_] 1Br is obtained by the reaction of [Cl(CH_2_CF_3_)_2_][Sb(OTeF_5_)_6_] 1Cl with CH_2_BrCF_3_ (Scheme 1). After an excess of CH_2_BrCF_3_ is condensed onto a solution of 1Cl in SO_2_ClF at −196 °C, the reaction mixture is warmed up to −40 °C and then slowly warmed up further to 20 °C over the time of one hour. Removing all volatiles under reduced pressure yields 1Br as a colorless powder (78%). The bromonium salt is stable at room temperature for hours but decomposes overnight in an SO_2_ClF solution.

**Fig. 1:**
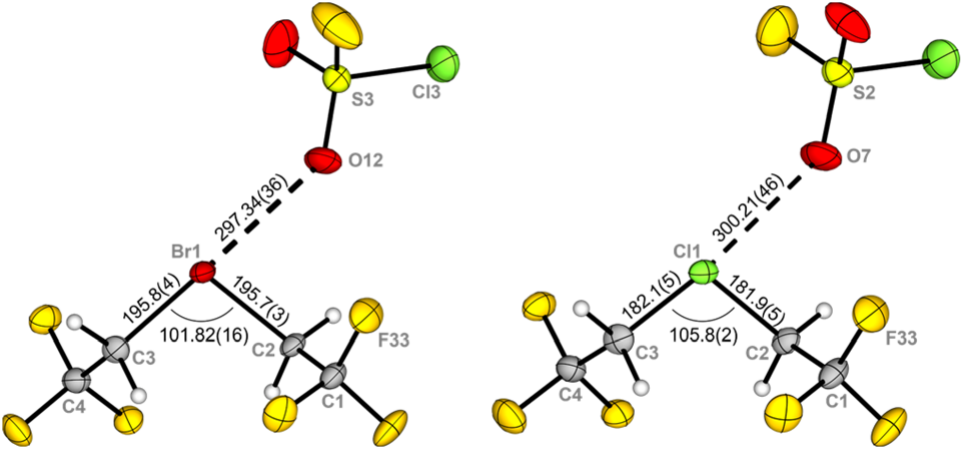
Molecular structure of the bromonium cation in 1Br (left) and the isostructural chloronium cation in 1Cl in the solid state. Two of the three co-crystalized solvent molecules SO_2_ClF and the anion [Sb(OTeF_5_)_6_]^−^ are omitted for clarity. Displacement ellipsoids set at 50% probability. Selected bond lengths [pm] and angles [°]: 1Br C1–C2 151.4(5), C3–C4 151.4(5), C1–C2–C3–C4 140.4(3); 1Cl C1–C2 1.507(7), C3–C4 1.517(7), C1–C2–C3–C4 141.0(4). For crystallographic details see ESI.†

The ^1^H and ^13^C{^1^H,^19^F} NMR spectra of 1Br (*δ*(^1^H) = 5.65 ppm, ^1^*J*_C–H_ = 164 Hz; *δ*(^13^C{^1^H,^19^F}) = 54.7 ppm, 120.1 ppm) show the expected low-field shift of the signals of the methylene group compared to the starting material CH_2_BrCF_3_ (*δ*(^1^H) = 4.08 ppm; *δ*(^13^C{^1^H}) = 25.2 ppm, 123.6 ppm). The ^19^F NMR spectrum shows the triplet signal of the CF_3_ group in the bromonium cation at −66.1 ppm and the signal of the collapsed AB_4_ spin system of the OTeF_5_ groups in the antimonate anion at −41.9 ppm. The complex signal of the antimonate anion has previously been discussed in detail in the literature.^12^ Single crystals of 1Br suitable for X-ray diffraction could be obtained by dissolving the compound in SO_2_ClF and condensing isobutane at −196 °C onto the frozen solution. The layered sample was immediately stored in a −80 °C freezer. Compound 1Br crystallizes in the triclinic space group *P*1̄ (Fig. 1). It marks the first obtained molecular structure of an acyclic dialkyl bromonium salt, with the only other reported dialkyl structure showing a three-membered cyclic bromonium moiety connected to 2,2′-biadamantane.^13^ The anion in 1Br, which has already been described in detail in literature,^12,14^ consists of an antimony center octahedrally coordinated by six OTeF_5_ moieties. The [Br(CH_2_CF_3_)_2_]^+^ cation forms a halogen bond with one of the two σ-holes on the bromine atom and a co-crystallized SO_2_ClF molecule (d(Br1–O12) = 297.34(36) pm). A second Br1–O8 bond length below the sum of their van-der-Waals radii (337 pm) with 323.01(36) pm is observed, but does not appear in a specific direction. The CF_3_ groups point in opposite directions with a torsion angle C1–C2–C3–C4 of 140.4(3)°. The C2–Br1–C3 bond angle is 101.82(16)° and the C–Br bond lengths are 195.8(4) pm and 195.7(3) pm and are in the range of C–Br distances in bromofluoro alkanes.^15^ Our group has previously reported the molecular structure of the lighter homolog, the chloronium salt 1Cl,^10^ which crystallized in the same space group (*P*1̄) but in a different unit cell than 1Br. However, while further studying the chloronium salt, a coincidence led to the isolation of colorless crystals of 1Cl. This newly recorded molecular structure of 1Cl is isostructural to that of 1Br (Fig. 1). Compared to the Cl–C bond lengths (182.1(5) pm and 181.9(5) pm) in the chloronium cation, the Br–C bond lengths are elongated by about 14 pm.

The halogen oxygen distances between the halonium cation and the SO_2_ClF molecules (1Cl: 300.21(46) pm and 314.49(44) pm) are in a similar range. The co-crystalized molecules of SO_2_ClF can be removed under reduced pressure. The absence of solvent is observed in the IR spectrum of the halonium salts (Fig. S24†). Due to their structural similarities, 1Cl, 1Br and the recently reported iodonium salt [I(CH_2_CF_3_)_2_][Sb(OTeF_5_)_6_] 1I ^10^ show comparable signals in the IR spectra. Signals corresponding to the halonium cations experience a slight red-shift with increasing sizes of the central halogen atom (Fig. S25†).

**Scheme 2:**
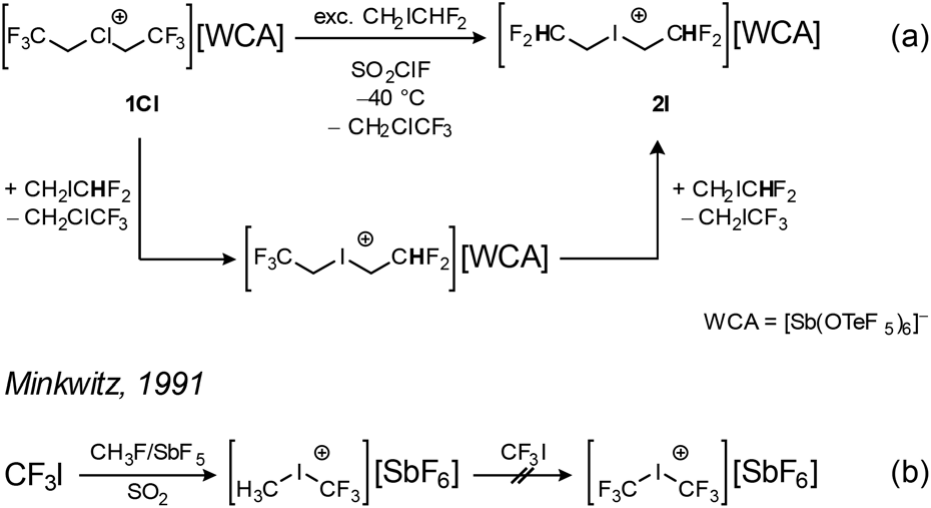
Synthesis of the iodonium salt 2I (a) and the asymmetric iodonium salt [I(CH_3_)(CF_3_)][SbF_6_] (b).

With the successful isolation of the halonium ions ([X(CH_2_CF_3_)_2_]^+^ X = Cl, Br, I), we were interested in the synthesis of differently substituted fluoroethyl halonium salts. To achieve that, we reacted the chloronium salt 1Cl with an excess of CH_2_ICHF_2_ in SO_2_ClF. The reaction does not yield the asymmetric iodonium cation [I(CH_2_CF_3_)(CH_2_CHF_2_)]^+^ but the symmetric [I(CH_2_CHF_2_)_2_]^+^ cation (Scheme 2a). Most likely, the asymmetric cation is formed *in situ* but is able to selectively transfer the less fluorinated alkyl chain CH_2_CHF_2_ to another molecule of CH_2_ICHF_2_, yielding the symmetric cation in 2I (91%). This result stands in contrast to the work of Minkwitz on fluorinated dialkyl iodonium salts, who was able to isolate the asymmetric salt [I(CF_3_)(CH_3_)][MF_6_] (M = As, Sb) (Scheme 2b).^16^ The product is obtained by methylation of CF_3_I, which is used in excess, by the strong methylation system CH_3_F/SbF_5_ in SO_2_. In this case, the CF_3_ group cannot be transferred from the asymmetric iodonium cation, as the resulting CH_3_I is more nucleophilic than CF_3_I, preventing the formation of a symmetric cation.

The ^1^H and ^13^C{^1^H,^19^F} NMR spectra of 2I show the signals of the methylene group at *δ*(^1^H) = 5.16 ppm and *δ*(^13^C{^1^H}) = 37.4 ppm and the signals of the CHF_2_ group at *δ*(^1^H) = 6.42 ppm and *δ*(^13^C{^1^H}) = 109.9 ppm. The ^19^F NMR spectrum shows the doublet of triplet signal of the CHF_2_ group at −109.8 ppm with coupling constants of ^2^*J*_F–H_ = 16.7 Hz and ^3^*J*_F–H_ = 1.8 Hz and the signal of the collapsed AB_4_ spin system of the OTeF_5_ groups in the antimonate anion at −41.9 ppm.

**Fig. 2:**
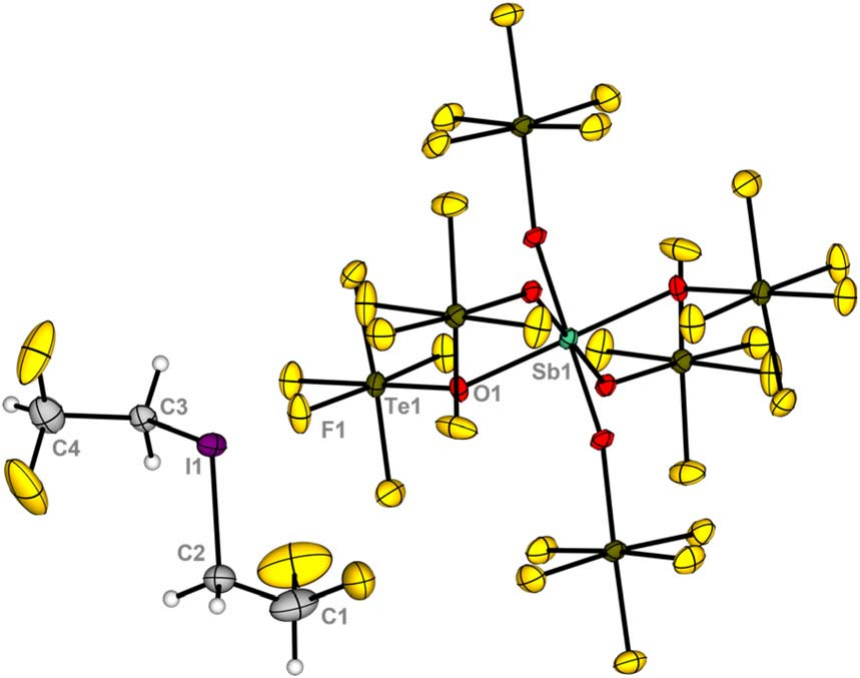
Molecular structure of the iodonium salt 2I in the solid state. Displacement ellipsoids set at 50% probability. For crystallographic details see ESI.†

Single crystals of 2I suitable for X-ray diffraction could be obtained by dissolving the compound in SO_2_ClF, layering the solution with *n*-pentane and storing the sample at −80 °C. Compound 2I crystallizes in the triclinic space group *P*1̄ (Fig. 2). The cation is disordered in the structure, preventing an accurate analysis of the bond length and angles.

In addition to the partially fluorinated diethyl halonium salts, we were interested in the synthesis of dipropyl derivatives. Our first attempts focused on the oxidation of 2-chloro-1,1,1,3,3,3-hexafluoropropane CHCl(CF_3_)_2_ to obtain a highly fluorinated diisopropyl chloronium salt. Unfortunately, CHCl(CF_3_)_2_ shows a high resistance towards strong oxidizers. While the xenonium salt [XeOTeF_5_][Sb(OTeF_5_)_6_] is able to oxidize the chlorine atom of CH_2_ClCF_3_, it does not in the case of CHCl(CF_3_)_2_ to yield the desired chloronium salt. Similar limitations have been observed by Schrobilgen, who showed that [XeOTeF_5_][Sb(OTeF_5_)_6_] is able to oxidize CFCl_3_ and CF_2_Cl_2_, yielding chlorofluoro carbocations, but is unreactive towards the higher fluorinated CF_3_Cl.^17^ Therefore, we replaced one electron-withdrawing CF_3_ group in CHCl(CF_3_)_2_ with a CH_3_ group. With this adjustment, the xenonium salt [XeOTeF_5_][Sb(OTeF_5_)_6_] oxidizes the Cl atom in 2-chloro-1,1,1-trifluoropropane CHCl(CH_3_)(CF_3_), to yield a highly reactive, temperature-sensitive colorless solid. Upon condensing CHCl(CH_3_)(CF_3_) onto the characteristically yellow xenonium salt solution in SO_2_ClF and warming the mixture up to −60 °C, discoloration and the precipitation of the product is observed. The low solubility in SO_2_ClF at low temperatures prevented the direct observation of the product *via* low temperature NMR spectroscopy. The high reactivity of the product led us to believe that a novel chloronium salt was obtained in this reaction.

**Fig. 3:**
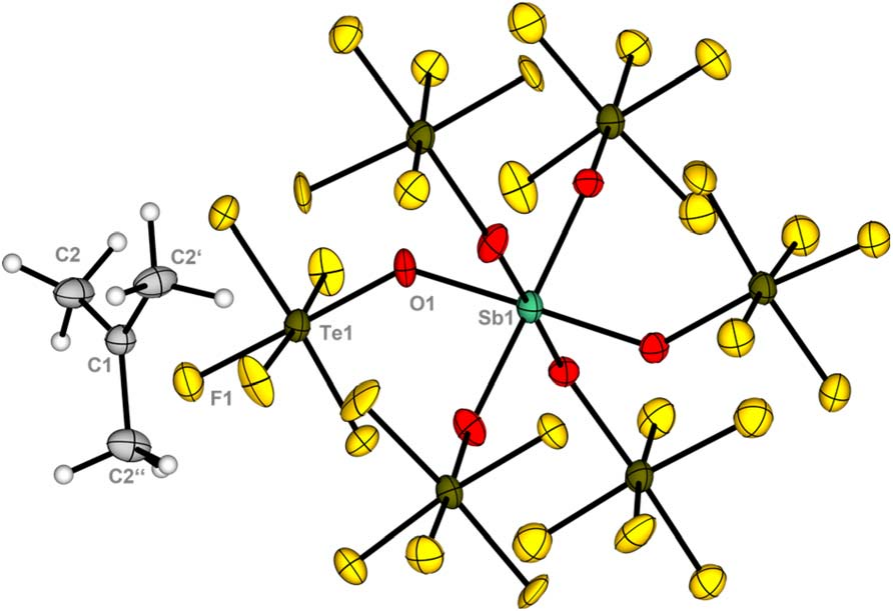
Molecular structure of the *tert*-butyl salt 3 in the solid state. Displacement ellipsoids set at 50% probability. For crystallographic details see ESI.†

The crystallization of the product presented significant challenges, as no suitable crystals for X-ray diffraction could be obtained from a solution of SO_2_ClF. The previously reported method for crystallizing fluorinated alkyl chloronium salts was unsuccessful in this instance.^10^ However, when an SO_2_ClF solution of the product was layered with isobutane, colorless crystals were isolated, which were identified as the *tert*-butyl salt [C_4_H_9_][Sb(OTeF_5_)_6_] 3. The compound crystalizes in the trigonal space group *R*3 (Fig. 3). The *tert*-butyl cation shows a trigonal planar structure with C–C bond lengths of 1.445(9) pm and C–C–C bond angles of 119.99(3)°. The obtained values are in good agreement with previously reported structures of the *tert*-butyl cation.^18–20^

The activation of alkanes confirms the exceptionally high reactivity previously reported for fluorinated dialkyl chloronium salts or highly fluorinated carbocations like the perfluoro trityl cation.^21^ For example, the chloronium salt 1Cl has been used to activate *n*-pentane, *n*-butane and cyclohexane to yield tertiary carbocations stabilized by the weakly coordinating [Sb(OTeF_5_)_6_]^−^ anion.^10^ Interestingly, isobutane showed no reactivity towards 1Cl, indicating a higher reactivity of the newly obtained chloronium salt.

As the direct characterisation of the product resulting from the oxidation of CHCl(CH_3_)(CF_3_) was unsuccessful, we added an electrophile scavenger to the obtained suspension to explore its alkylation ability. Pentafluoropyridine was chosen in this case as it was shown before that it reacts with the dimethyl chloronium salt [Cl(CH_3_)_2_][Al(OTeF_5_)_4_] and 1Cl to form room temperature stable pyridinium salts.^10,22^ Upon addition of a few drops of the pyridine to the cold suspension, a color change to dark red, indicating the formation of a pyridinium ion, was observed. The reaction mixture was warmed up to room temperature and all volatiles were removed under reduced pressure.

**Fig. 4:**
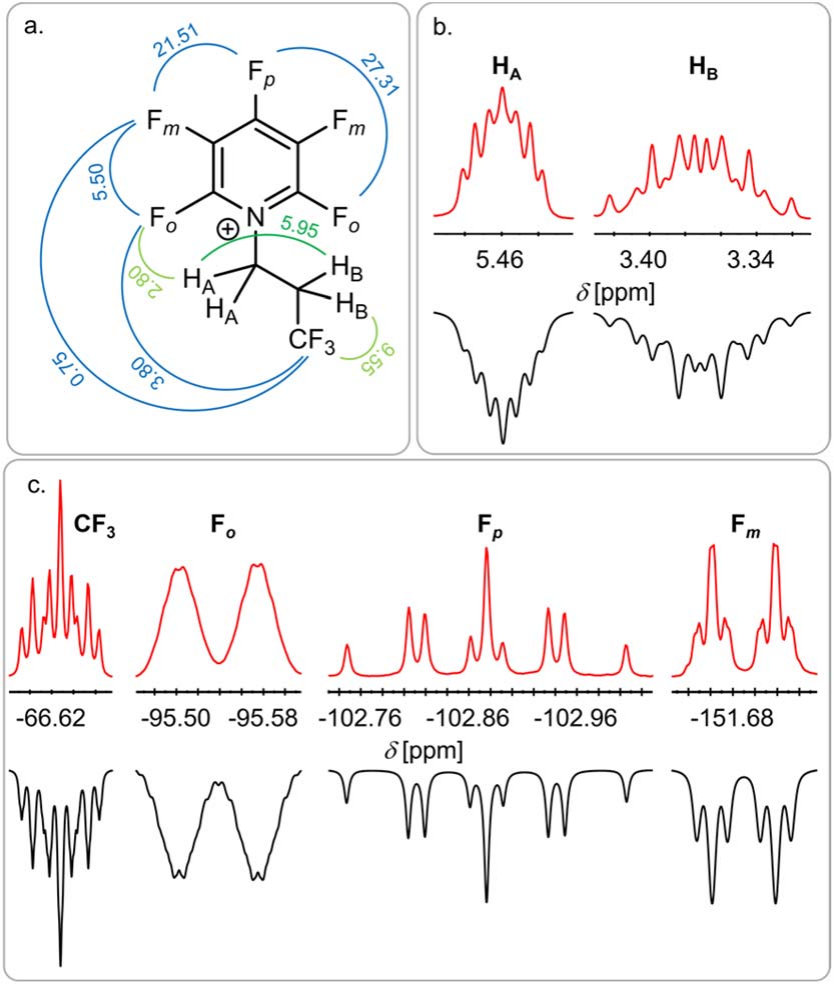
Structure of the obtained pyridinium ion with the observed coupling constants in Hz (a) and the corresponding signals in the ^1^H NMR spectrum (b) and the ^19^F NMR spectrum (c). Red: experimental spectrum, black: simulated spectrum. The full spectrum can be found in the ESI (Fig. S13).†

To our surprise, the NMR analysis of the reaction product shows the formation of the pyridinium salt [F_5_C_5_N(CH_2_CH_2_CF_3_)][Sb(OTeF_5_)_6_] 4 (97%), which does not contain a trifluoro isopropyl but rather a trifluoro *n*-propyl (CH_2_CH_2_CF_3_) motive (Fig. 4). The two H_A_ atoms of the CH_2_ group connected to the nitrogen atom show the characteristic low field shift of alkylated pentafluoropyridine.^10,22^ The splitting pattern of the signal is due to a coupling of H_A_ to H_B_ and the *ortho*-fluorine atoms F_o_ on the pyridine ring, leading to a triplet of triplets (^3^*J*_H–H_ = 5.95 Hz, ^4^*J*_H–F_ = 2.8 Hz). Furthermore, a coupling of the CF_3_ group to H_B_ (^3^*J*_H–F_ = 9.55 Hz) and a long-range coupling to F_o_ (^6^*J*_F–F_ = 3.8 Hz) and F_m_ (^7^*J*_F–F_ = 0.75 Hz) is observed.

The obtained data lead us to believe that the reaction product of [XeOTeF_5_][Sb(OTeF_5_)_6_] and CHCl(CH_3_)(CF_3_) is not the symmetric isopropyl but rather an *n*-propyl substituted chloronium salt. Quantum-chemical calculations have been performed to explain the formation of this elusive chloronium ion.

**Fig. 5:**
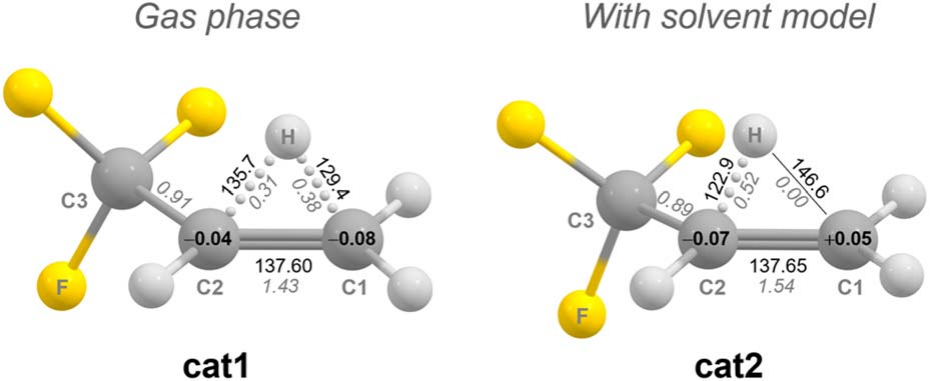
Calculated structures with selected bond lengths, bond orders (italics) and atomic Mulliken charges of the carbocationic intermediate cat1 in the gas phase and cat2 with a solvent model applied formed by the oxidative halide abstraction of CHCl(CH_3_)(CF_3_) with [XeOTeF_5_][Sb(OTeF_5_)_6_] (B3LYP-D3(BJ)/cc-pVTZ). Bond lengths are given in pm.

When optimizing the structure of the secondary carbocation [CH(CH_3_)(CF_3_)]^+^ that should result from the initial oxidation of CHCl(CH_3_)(CF_3_), we observe a migration of one hydrogen atom of the CH_3_ group towards the secondary carbon atom cat1 (Fig. 5). We assume the CF_3_ group destabilizes the positive charge at the secondary carbon atom. As a result, the C1–C2 bond shows an increased double bond character (C1–C2 bond length 137.6 pm, NBO bond order 1.43). The hydrogen atom is positioned above the C1–C2 bond, resulting in comparable C1–H and C2–H bonds lengths and NBO bond orders. The C1 and C2 atoms possess slightly negative Mulliken charges, allowing to describe cat1 as a trifluoropropene molecule, with a proton coordinated to the C1–C2 bond.

When a solvent model is applied (*ε* = 100), the hydrogen atom shifts further towards the secondary carbon atom cat2. Therefore, the C1–C2 double bond character is increased (NBO bond order C1–C2: 1.54), whereas the C1–H NBO bond order drops to zero. The terminal atom (C1) is now slightly positively charged. Further computational details and the results of a QTAIM analysis for cat1 and cat2 can be found in the ESI.†

In general, aliphatic carbocations can act as strong Brønsted acids and eliminate a proton through double bond formation in the presence of a nucleophile.^23^ However, nonfluorinated aliphatic carbocations can be stabilized in the gas phase or the condensed phase in weakly nucleophilic solvents and with weakly coordinating anions (WCAs).^10,18–20,24^

In the present case, the CF_3_ group increases the Brønsted acidity of the initial secondary carbocation to the point that deprotonation of the CH_3_ group occurs even without a nucleophilic partner, followed by the subsequent coordination of the proton to the newly formed double bond.

**Scheme 3:**
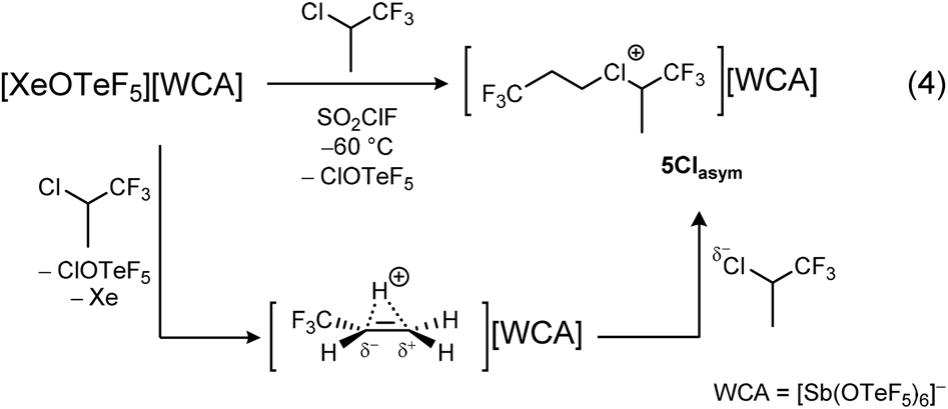
Synthesis of the asymmetric chloronium salt 5Cl_asym_.

Upon formation, cat2 can readily react with the negative polarized chlorine atom in CHCl(CH_3_)(CF_3_), with a reaction enthalpy of −106 kJ mol^−1^, leading to the formation of the asymmetric chloronium salt [Cl(CH_2_CH_2_CF_3_)(CH(CH_3_)(CF_3_))][Sb(OTeF_5_)_6_] 5Cl_asym_ (Scheme 3). The asymmetric ion in 5Cl_asym_ is energetically favoured by 5 kJ mol^−1^ (with solvent model 21 kJ mol^−1^) compared to the symmetric diisopropyl [Cl(CH(CH_3_)(CF_3_))_2_]^+^ ion. The earlier described reaction of 5Cl_asym_ with NC_5_F_5,_ yielding exclusively the *n*-propyl substituted pyridinium salt 4 (Fig. 4), can then be explained by the energetically favoured transfer of the *n*-propyl group (−122 kJ mol^−1^) compared to the transfer of the isopropyl group (−97 kJ mol^−1^).

**Scheme 4:**
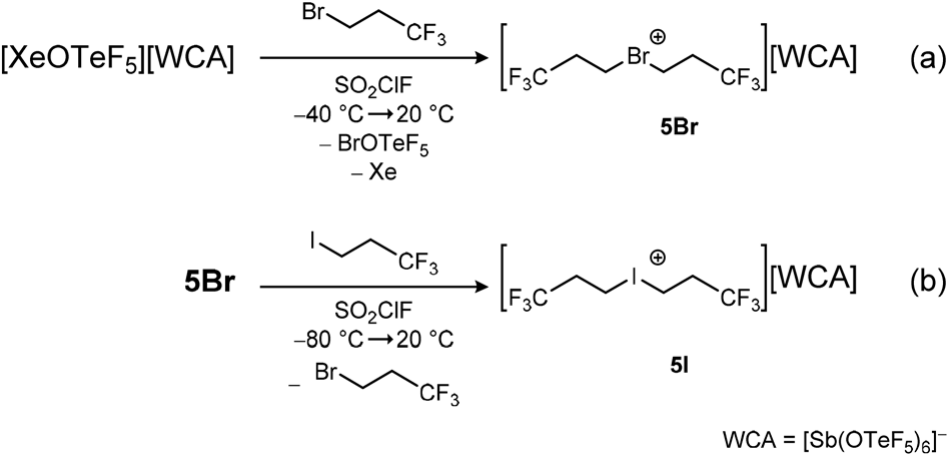
Synthesis of the bromonium salt 5Br (a) and the iodonium salt 5I (b).

While the fluorinated asymmetric dipropyl chloronium salt 5Cl_asym_ could not be isolated due to its high reactivity and low stability, we were interested in the synthesis of more stable fluorinated symmetric dipropyl bromonium and iodonium salts. The reaction of the xenonium salt [XeOTeF_5_][Sb(OTeF_5_)_6_] and 1-bromo-3,3,3-trifluoropropane CH_2_BrCH_2_CF_3_ in SO_2_ClF yields a dark red solution, indicating the formation of the side product BrOTeF_5_, which is described in literature as a ruby red liquid (Scheme 4a).^25^ Removing all volatiles under reduced pressure and washing the product with *n*-pentane yields the bromonium salt [Br(CH_2_CH_2_CF_3_)_2_][Sb(OTeF_5_)_6_] 5Br as an off-white powder in good yields (89%). The compound is stable at room temperature for hours but decomposes overnight. The ^1^H and ^13^C{^1^H,^19^F} NMR spectra show the characteristic low-field shift of the signals corresponding to the CH_2_ group connected to the bromine atom upon halonium ion formation (*δ*(^1^H) = 5.46 ppm and *δ*(^13^C{^1^H,^19^F}) = 34.0 ppm, 59.0 ppm, 123.0 ppm). A shift of Δ*δ* 1.56 ppm in the ^1^H and 38.8 ppm in the ^13^C{^1^H,^19^F} NMR spectra of the obtained product compared to the starting material indicates the successful halonium salt formation, with similar shifts being observed in the [Br(CH_2_CF_3_)_2_]^+^ ion.

The iodonium salt [I(CH_2_CH_2_CF_3_)_2_][Sb(OTeF_5_)_6_] 5I is obtained by the reaction of 5Br with 1,1,1-trifluoro-3-iodopropane in SO_2_ClF (Scheme 4b). Compound 5I is obtained as a dark red, room temperature stable solid in good yields (88%). The reaction of 5Br with an iodofluoroalkane shows its ability to act as a strong alkylating agent, transferring the trifluoropropyl group to weakly basic substrates.

## Conclusions

In conclusion, we report on the synthesis and characterization of the fluorinated diethyl and dipropyl halonium salts 1Br, 2I, 5Br and 5I. We obtained the molecular structures of 1Br, 2I, and the previously reported 1Cl in the solid state. Notably, 1Br is of particular interest as it represents the first structure of an acyclic dialkyl bromonium salt. Furthermore, we obtained a highly reactive product from the reaction of 2-chloro-1,1,1-trifluoropropane with [Xe(OTeF_5_)][Sb(OTeF_5_)_6_]. Due to the compound's thermal instability and low solubility, direct characterization was not possible. However, its reactivity towards isobutane and NC_5_F_5_, along with quantum-chemical calculations, suggests the formation of the asymmetric fluorinated dialkyl chloronium ion 5Cl_asym_.

## Author contributions

L. F. designed and performed the experiments, analyzed the data and prepared the manuscript. M. H. L. performed experiments. A. W. measured and refined the crystal structures and revised the manuscript. C. M. performed quantum-chemical calculations on the reactivity and structure of the asymmetric chloronium ion. S. R. supervised the project and revised the manuscript.

## Conflicts of interest

There are no conflicts to declare.

## Supplementary Material

SC-OLF-D5SC03756E-s001

SC-OLF-D5SC03756E-s002

## Data Availability

Additional details regarding experimental methods and experimental data are given in the ESI.†
