# Supplementary material for: On the synthesis and structure of reactive halonium ions
Source: Chem Sci. 2025 Jun 19;16(29):13229–34. doi: 10.1039/d5sc03756e (PMC12198972; doi:10.1039/d5sc03756e)
Supplement: SC-016-D5SC03756E-s001 [file SC-016-D5SC03756E-s001.pdf]

Supplementary Information (SI) for Chemical Science.
This journal is © The Royal Society of Chemistry 2025

Supporting Information

for

On the Synthesis and Structure of Reactive Halonium Ions

Lukas Fischer,[a] Michael H. Lee,[a] Anja Wiesner,[a] Carsten Müller,[a] Sebastian Riedel*[a]

[a] Fachbereich Biologie, Chemie, Pharmazie
Institut für Chemie und Biochemie – Anorganische Chemie
Freie Universität Berlin
Fabeckstraße 34/36, 14195 Berlin (Germany)
E-mail: s.riedel@fu-berlin.de

Table of contents

1. Experimental section S2
2. NMR spectra S8
3. IR spectra S20
4. Raman spectra S23
5. Crystal data S24
6. Quantum-chemical calculations S25
7. References S33

1 Experimental section

General procedures and materials

All experiments were performed under exclusion of moisture and oxygen using standard Schlenk techniques. Solids were handled in a glovebox under an argon atmosphere (O_2 < 0.5 ppm, H_2O < 0.5 ppm). Solvents were dried using an MBraun SPS-800 solvent system (CH_2Cl_2, *n*-pentane) or CaH_2 (SO_2ClF) before use and stored over 3 or 4 Å molecular sieves. $CHCl(CH_3)(CF_3)$,[1] $[Cl(CH_2CF_3)_2][Sb(OTeF_5)_6]$,[2] $[I(CH_2CF_3)_2][Sb(OTeF_5)_6]$,[2] $Xe(OTeF_5)_2$[3] and $Sb(OTeF_5)_3$[4] were prepared as described elsewhere. All other reagents were purchased from standard commercial suppliers and used as received. IR spectra were measured on neat solid samples at room temperature inside a glovebox under an argon atmosphere using a Bruker ALPHA FTIR spectrometer with a diamond ATR attachment with 32 scans and a resolution of 4 cm^{-1}. IR spectra were processed using OPUS 7.5 and Origin 9.1.[5] was used for their graphical representation. NMR spectra were recorded on a JEOL 400 MHz ECS or ECZ spectrometer. Crystal data were collected with MoKα radiation on a Bruker D8 Venture diffractometer with a CMOS area detector. Single crystals were picked at –40 °C under nitrogen atmosphere and mounted on a 0.15 mm micromount using perfluoroether oil. The structures were solved with the ShelXT[6] structure solution program using intrinsic phasing and refined with the ShelXL[7] refinement package using least squares minimizations by using OLEX2.[8] The program Diamond V4.6.8 was used for visualization.[9] CCDC 2453010, CCDC 2453011, CCDC 2453012 and CCDC 2453013 contain the supplementary crystallographic data for this paper. These data are provided free of charge by The Cambridge Crystallographic Data Centre. Crystal data and other details of the structure analyses are summarized in chapter 5 (Crystal data). Suitable crystals for X-ray diffraction studies were obtained as indicated in the corresponding experimental entry.

Synthesis of CH_2BrCF_3

The compound was obtained by applying a modified method to synthesize short-chain bromofluoroalkanes.[1]

A mixture of 1,1,1-trifluoroethyl nonaflate (10.8 g, 28.37 mmol, 5 eq.), 12-crown-4 (1.0 g, 568 mmol, 1. eq) and acetylacetone (100 mL) is placed under an argon atmosphere in a 250 ml round-bottomed Schlenk flask equipped with a reflux condenser cooled to –20 °C connected to a cooling trap cooled to –80 °C to prevent the condensation of water inside the condenser. Via a funnel, LiBr (9.85 g, 113.5 mmol, 20 eq.) is added slowly to facilitate fast dissolution of the salt. The mixture is heated to 100 °C for 15 h. After the reaction is completed, it is allowed to cool down to room temperature. Afterwards, a second cooling trap cooled to –196 °C is connected to the first cooling trap and dynamic vacuum is applied to the whole apparatus. The crude product is collected in the second cooling trap. Purification by Spaltrohr distillation yielded the product as a colorless, volatile liquid (3.54 g, 21.4 mmol, 74%).

^{1}H NMR (400 MHz, SO_2ClF, ext. [D6]acetone, 19.2 °C): δ = 4.08 (q, 2H, $-CH_2Br$, $^1J_{C\text{-}H}$ = 154.0 Hz, $^3J_{H\text{-}F}$ = 8.95 Hz) ppm.

^{13}C{^{1}H,^{19}F} NMR (101 MHz, SO_2ClF, ext. [D6]acetone, 19.3 °C): δ = 25.2 ($-CH_2-$), 123.6 ($-CF_3$) ppm.

^{19}F NMR (377 MHz, SO_2ClF, ext. [D6]acetone, 19.1 °C): δ = –70.2 (t, 3F, $-CF_3$, $^1J_{C\text{-}F}$ = 273.6 Hz, $^3J_{F\text{-}H}$ = 8.95 Hz) ppm.

Synthesis of $[Br(CH_2CF_3)_2][Sb(OTeF_5)_6]$ (**1Br**)

$[Cl(CH_2CF_3)_2][Sb(OTeF_5)_6]$ **1Cl** (305 mg, 0.174 mmol, 1 eq.) was weighed into a Schlenk tube with a greaseless Teflon stopcock. Afterwards, SO_2ClF (0.5 mL) was condensed onto the solid at –196 °C. The Schlenk tube was warmed up to room temperature and stirred until all solids were dissolved. The solution was cooled to –196 °C and an excess of CH_2BrCF_3 (0.1 mL) was condensed onto the frozen solution and warmed up to –40 °C. Over the time of 1 hour, the solution was warmed up to 20 °C. All volatile compounds of this now red/orange solution were removed under reduced pressure, yielding **1Br** as a colorless solid (243 mg, 0.135 mmol, 78%).

Crystals of **1Br** were obtained by dissolving the salt in 0.5 mL SO_2ClF, condensing isobutane on top of the frozen solution at -196 °C and immediately storing the still solid sample in a –80 °C freezer.

^{1}H NMR (400 MHz, SO_2ClF, ext. [D6]acetone, 18.5 °C): δ = 5.65 (q, 4H, -CH_2-, $^1J_{C\text{-}H}$ = 164.0 Hz, $^3J_{H\text{-}F}$ = 7.8 Hz) ppm.

^{13}C{^{1}H,^{19}F} NMR (101 MHz, SO_2ClF, ext. [D6]acetone, 19.2 °C): δ = 54.7 (-CH_2-), 120.1 (-CF_3) ppm.

^{19}F NMR (377 MHz, SO_2ClF, ext. [D6]acetone, 18.5 °C): δ = –41.9 (m, 30F, -$OTeF_5$), –66.1 (t, 6F, -CF_3, $^1J_{C\text{-}F}$ = 116.6 Hz, $^3J_{F\text{-}H}$ = 7.8 Hz) ppm.

IR (ATR, 20 °C): $\tilde{\nu}$ = 3063 (w), 2992 (w), 1413 (w), 1303 (m), 1267 (m), 1233 (m), 1159 (m), 1148 (m) 1078 (m), 857 (vs), 716 (vs), 691 (vs), 652 (m), 627 (m), 522 (w), 464 (m), 444 (m) cm^{-1}.

Synthesis of $[I(CH_2CHF_2)_2][Sb(OTeF_5)_6]$ (**2I**)

Freshly prepared $[Cl(CH_2CF_3)_2][Sb(OTeF_5)_6]$ **1Cl** ($Sb(OTeF_5)_3$: 85.0 mg, 0.101 mmol; $Xe(OTeF_5)_2$: 123 mg, 0.203 mmol) was dissolved in SO_2ClF (0.5 mL) in a Schlenk tube with a greaseless Teflon stopcock. Afterwards, the solution was cooled to –80 °C and an excess of CH_2ICHF_2 (0.1 mL) was added via a syringe to the solution and warmed up to room temperature over the time of 1 hour. Afterwards, the solution was cooled to –10 °C and ca. 3 mL pentane was added to the solution, resulting in the precipitation of a dark red solid. The liquid phase was removed via a syringe and the obtained solid was dried under reduced pressure, yielding **2I** as a dark red powder (161 mg, 0.092 mmol, 91%).

Crystals of **2I** were obtained by dissolving the salt in 0.5 mL SO_2ClF, layering the solution with *n*-pentane and storing the sample in a –40 °C freezer.

^{1}H NMR (400 MHz, SO_2ClF, ext. [D6]acetone, 19.1 °C): δ = 6.42 (m, 2H, -CHF_2), 5.16 (m, 4H, -CH_2-) ppm.

^{13}C{^{1}H} NMR (101 MHz, SO_2ClF, ext. [D6]acetone, 18.5 °C): δ = 37.4 (-CH_2-), 109.9 (-CHF_2) ppm.

^{19}F NMR (377 MHz, SO_2ClF, ext. [D6]acetone, 19.1 °C): δ = –41.9 (m, 30F, -$OTeF_5$), –109.8 (m, 4F, -CHF_2) ppm.

IR (ATR, 20 °C): $\tilde{\nu}$ = 3053 (w), 2987 (w), 1632 (w), 1600 (w), 1550 (w), 1467 (w), 1419 (w), 1401 (w), 1349 (m), 1287 (w), 1259 (w), 1234 (w), 1218 (w), 1176(m), 1137(m), 1104 (w), 1049 (w), 1020 (w), 957 (w), 851 (vs), 715 (vs), 690 (vs), 637 (s), 515 (w), 464 (s) cm^{-1}.

Reaction of $[XeOTeF_5][Sb(OTeF_5)_6]$ with $CHCl(CH_3)(CF_3)$

$Sb(OTeF_5)_3$ (50.0 mg, 0.06 mmol, 1 eq.) was weighed into a Schlenk tube with a greaseless Teflon stopcock. Afterwards, SO_2ClF (0.5 mL) was condensed onto the solid at –196 °C. The Schlenk tube was warmed up to –80 °C. To the colorless solution, $Xe(OTeF_5)_2$ (73.0 mg, 0.12 mmol, 2 eq.) was added via a funnel. The mixture was allowed to warm to 0 °C over the time of 1 hour. The now yellow solution was warmed up to 20 °C for 5 min and then cooled to –196 °C. Onto the frozen solution, an excess of 2-chloro-1,1,1-trifluoropropane $CHCl(CH_3)(CF_3)$ (0.1 mL) was condensed and warmed up to –60 °C. The reaction mixture was stirred for 1 h at that temperature. Slowly, the solution turns from yellow to colorless, while a colorless solid precipitates. All volatile compounds were removed under reduced pressure at –80 °C, yielding a colorless, temperature-sensitive solid.

Crystal grows of $[C_4H_9][Sb(OTeF_5)_6]$ (**3**)

A suspension of the product obtained from the reaction of $[XeOTeF_5][Sb(OTeF_5)_6]$ with $CHCl(CH_3)(CF_3)$ was carefully warmed until all solid was dissolved and immediately frozen solid using liquid nitrogen. Afterwards, isobutane (1 mL) was condensed onto the frozen solution. The layered frozen solution was placed in a –80 °C freezer. After one day, colorless crystals of **3** had been obtained.

Synthesis of $[F_5C_5N(CH_2CH_2CF_3)][Sb(OTeF_5)_6]$ (**4**)

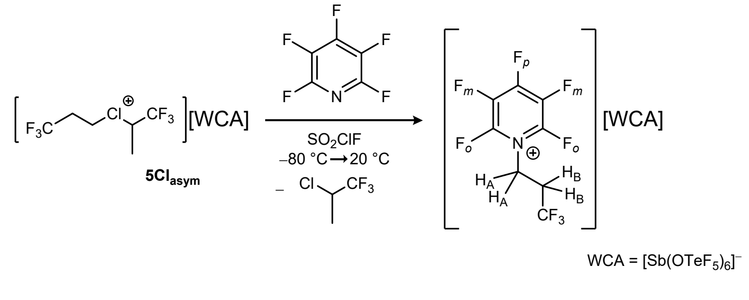

$Sb(OTeF_5)_3$ (50.0 mg, 0.0597 mmol, 1 eq.) was weighed into a Schlenk tube with a greaseless Teflon stopcock. Afterwards, SO_2ClF (0.5 mL) was condensed onto the solid at –196 °C. The

Schlenk tube was warmed up to –80 °C. To the colorless solution, $Xe(OTeF_5)_2$ (73.0 mg, 0.12 mmol, 2 eq.) was added via a funnel. The mixture was allowed to warm to 0 °C over the time of 1 hour. The now yellow solution was warmed up to 20 °C for 5 min and then cooled to –196 °C. Onto the frozen solution, an excess of $CHCl(CH_3)(CF_3)$ (0.1 mL) was condensed and warmed up to –100 °C over the time of 2 hours. The reaction mixture was allowed to warm up to –60 °C, yielding a pale yellow suspension. The suspension was cooled down to –80 °C and a few drops of pentafluoropyridine NC_5F_5 were added via a syringe, resulting in an immediate color change to orange and upon warming the reaction mixture up to room temperature, to dark red. Removing all volatiles under reduced pressure yields **4** as a dark red solid (105.6 mg, 58 mmol, 97%).

^{1}H NMR (400 MHz, SO_2ClF, ext. [D6]acetone, 20.5 °C): δ = 5.46 (tt, 2H, H_A, $^3J_{H\text{-}H}$ = 5.95 Hz, $^4J_{H\text{-}F}$ = 2.80 Hz), 5.46 (qt, 2H, H_B, $^3J_{H\text{-}F}$ = 9.55 Hz, $^3J_{H\text{-}H}$ = 5.95 Hz) ppm.

^{19}F NMR (377 MHz, SO_2ClF, ext. [D6]acetone, 20.5 °C): δ = –42.1 (m, 30F, -$OTeF_5$), −66.6 (ttt, 3F, -CF_3, $^3J_{F\text{-}H}$ = 9.55 Hz, $^6J_{F\text{-}H}$ = 3.80 Hz, $^7J_{F\text{-}H}$ = 0.75 Hz), –95.5 (m, 2F, F_o, $^3J_{F\text{-}F}$ = 5.5 Hz, $^4J_{F\text{-}H}$ = 2.80 Hz, $^6J_{F\text{-}H}$ = 3.80 Hz), −102.9 (tt, 1F, F_p, $^3J_{F\text{-}F}$ = 21.51 Hz, $^4J_{F\text{-}F}$ = 27.31 Hz), −151.7 (m, 2F, F_m, $^3J_{F\text{-}F}$ = 21.51 Hz, $^3J_{F\text{-}F}$ = 5.50 Hz, $^7J_{F\text{-}F}$ = 0.75 Hz) ppm.

Synthesis of $[Br(CH_2CH_2CF_3)_2][Sb(OTeF_5)_6]$ (**5Br**)

$Sb(OTeF_5)_3$ (90.0 mg, 0.107 mmol, 1 eq.) was weighed into a Schlenk tube with a greaseless Teflon stopcock. Afterwards, SO_2ClF (0.5 mL) was condensed onto the solid at –196 °C. The Schlenk tube was warmed up to –80 °C. To the colorless solution, $Xe(OTeF_5)_2$ (130.0 mg, 0.214 mmol, 2 eq.) was added via a funnel. The mixture was allowed to warm to 0 °C over the time of 1 hour. The now yellow solution was warmed up to 20 °C for 5 min and then cooled to –196 °C. Onto the frozen solution, an excess of 3-bromo-1,1,1-trifluoropropane $CH_2BrCH_2CF_3$ (0.1 mL) was condensed and warmed up to –40 °C. Over the time of 1 hour the solution was warmed up to 20 °C. All volatile compounds of this now colorless solution were removed under reduced pressure, yielding **5Br** as a colorless solid (164 mg, 0.0954 mmol, 89%).

^{1}H NMR (400 MHz, SO_2ClF, ext. [D6]acetone, 20 °C): δ = 5.46 (t, 4H, -Br-CH_2-, $^3J_{H\text{-}H}$ = 5.7 Hz), 3.59 (qt, 4H, -CH_2-, $^3J_{H\text{-}H}$ = 5.7 Hz, $^3J_{H\text{-}F}$ = 9.1 Hz) ppm.

^{13}C{^{1}H,^{19}F} NMR (101 MHz, SO_2ClF, ext. [D6]acetone, 19.9 °C): δ = 34.0 (-CH_2-), 59.0 (-Br-CH_2-), 123.0 (-CF_3) ppm.

^{19}F NMR (377 MHz, SO_2ClF, ext. [D6]acetone, 20 °C): δ = –41.9 (m, 30F, -$OTeF_5$), –66.3 (t, 6F, -CF_3, $^3J_{F\text{-}H}$ = 9.0 Hz) ppm.

IR (ATR, 20 °C): $\tilde{\nu}$ = 3065 (w), 3000 (w), 2951 (w), 1423 (m), 1393 (m), 1372 (m), 1298 (w), 1264 (m), 1245 (m), 1224 (m), 1204 (m), 1176 (m), 1148 (m), 1094 (m), 1030 (w), 860 (vs), 714 (vs), 691 (vs), 645 (m), 572 (m), 550 (m), 539 (m), 493 (s), 462 (m), 437 (m) cm^{-1}.

Raman (20 °C): $\tilde{\nu}$ = 2996 (m), 2956 (m), 710 (s), 661 (s), 645 (m), 542 (w), 499 (w), 436 (w), 399 (m), 333 (m), 307 (m), 237 (m), 138 (m), 106 (m) cm^{-1}.

Synthesis of $[I(CH_2CH_2CF_3)_2][Sb(OTeF_5)_6]$ (**5I**)

$[Br(CH_2CH_2CF_3)_2][Sb(OTeF_5)_6]$ **5Br** (70 mg, 0.0407 mmol, 1 eq.) was weighed into a Schlenk tube with a greaseless Teflon stopcock. Afterwards, SO_2ClF (0.5 mL) was condensed onto the solid at –196 °C. The Schlenk tube was warmed up to room temperature and stirred until all solids were dissolved. The solution was cooled to –80 °C and an excess of 1,1,1-trifluoro-3-iodopropane $CH_2ICH_2CF_3$ (0.1 mL) was added via a syringe. The now violet solution was warmed up to room temperature. After 20 min, ca. 3 mL of pentane was added to the solution, resulting in the precipitation of a dark red solid. The liquid phase was removed via a syringe and the obtained solid was dried under reduced pressure, yielding **5I** as a dark red powder (59 mg, 0.0334 mmol, 82%).

^{1}H NMR (400 MHz, SO_2ClF, ext. [D6]acetone, 20 °C): δ = 5.0 (t, 4H, -I-CH_2-, $^1J_{C\text{-}H}$ = 164.0 Hz, $^3J_{H\text{-}H}$ = 5.6 Hz), 3.54 (tq, 4H, -CH_2-, $^3J_{H\text{-}H}$ = 6.1 Hz, $^3J_{H\text{-}F}$ = 9.1 Hz) ppm.

^{13}C{^{1}H,^{19}F} NMR (101 MHz, SO_2ClF, ext. [D6]acetone, 21 °C): δ = 29.9 (-I-CH_2-), 33.8 (-CH_2-), 124.7 (-CF_3) ppm.

^{19}F NMR (377 MHz, SO_2ClF, ext. [D6]acetone, 20 °C): δ = –41.9 (m, 30F, -$OTeF_5$), –66.9 (t, 6F, -CF_3, $^1J_{C\text{-}F}$ = 276 Hz, $^3J_{F\text{-}H}$ = 9.2 Hz) ppm.

IR (ATR, 20 °C): $\tilde{\nu}$ = 3053 (w), 2993 (w), 2949 (w), 1426 (m), 1391 (m), 1369 (m), 1294 (m), 1256 (m), 1227 (m), 1211 (m), 1189 (m), 1166 (m), 1136 (m), 1083 (m), 1025 (m), 860 (vs), 717 (vs), 692 (vs), 644 (m), 626 (m), 566 (m), 551 (m), 463 (s), 430 (m), cm^{-1}.

Raman (20 °C): $\tilde{\nu}$ = 2987 (m), 2949 (m), 706 (s), 661 (s), 640 (m), 481 (w), 433 (w), 400 (m), 334 (m), 307 (m), 237 (m), 140 (m), 109 (m) cm^{-1}.

2 NMR spectra

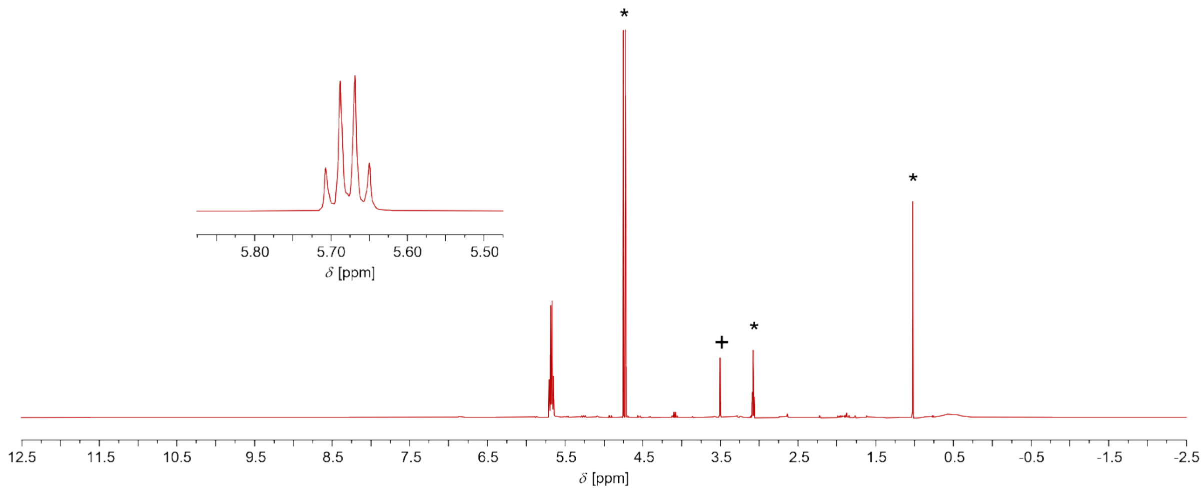

Figure S1. ^{1}H NMR spectrum (400 Hz, 18.5 °C, SO_2ClF, ext. [D6]acetone) of $[Br(CH_2CF_3)_2][Sb(OTeF_5)_6]$ **1Br**. Signals of the external standard are marked with (*). Unidentified impurity is marked with (+).

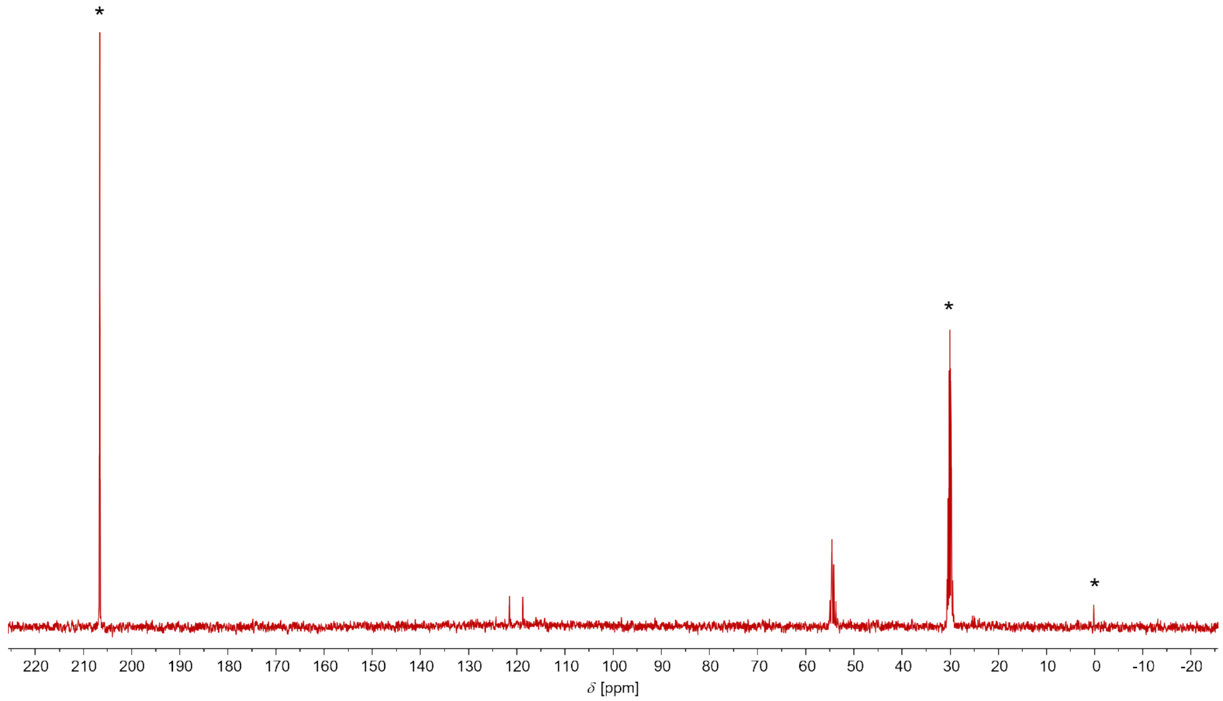

Figure S2. $^{13}C\{^{1}H\}$ NMR spectrum (101 Hz, 0 °C, SO_2ClF, ext. [D6]acetone) of $[Br(CH_2CF_3)_2][Sb(OTeF_5)_6]$ **1Br**. Signals of the external standard are marked with (*).

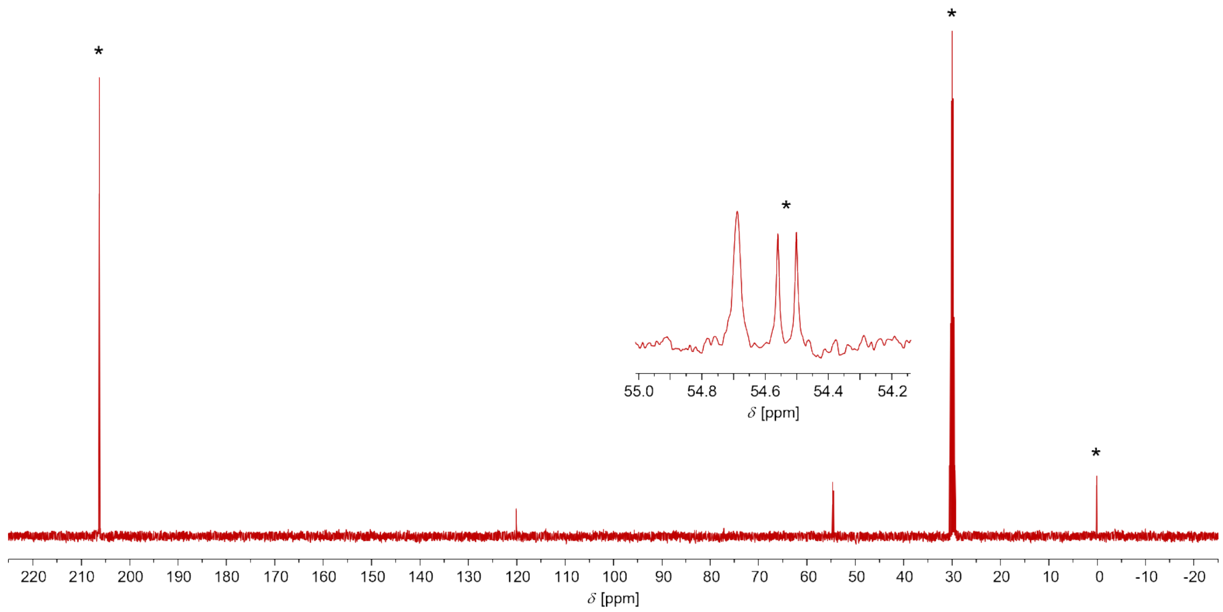

Figure S3. $^{13}C\{^{1}H,^{19}F\}$ NMR spectrum (101 Hz, 19.2 °C, SO_2ClF, ext. [D6]acetone) of $[Br(CH_2CF_3)_2][Sb(OTeF_5)_6]$ **1Br**. Signals of the external standard are marked with (*).

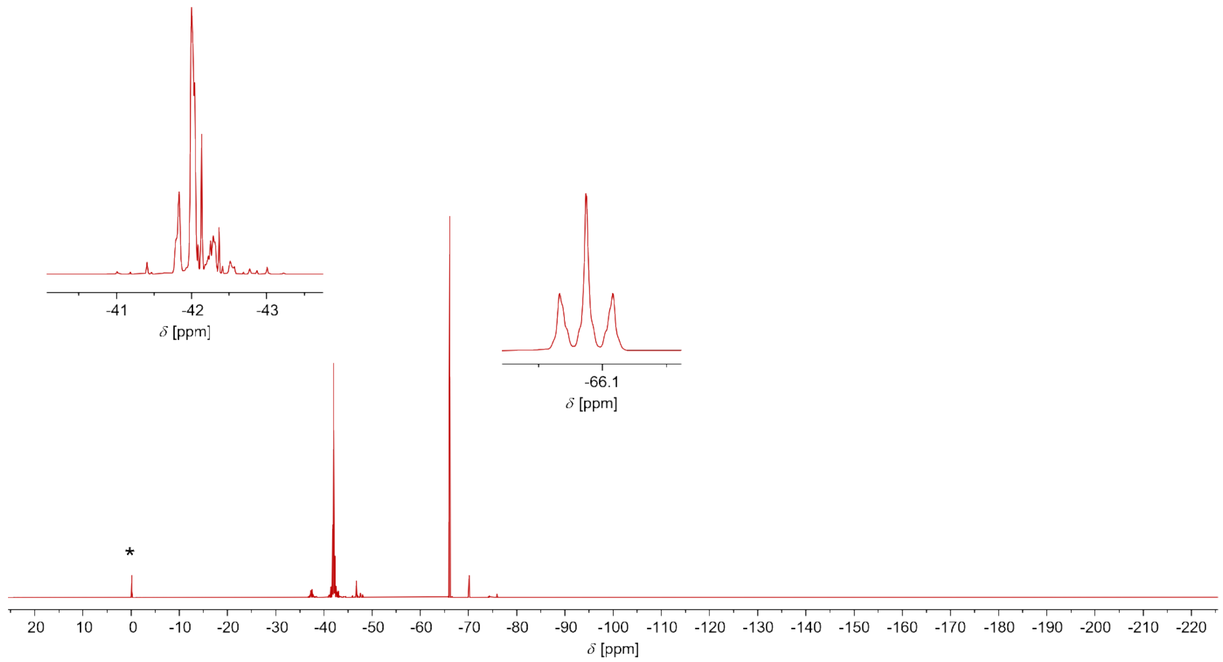

Figure S4. ^{19}F NMR spectrum (377 Hz, 18.5 °C, SO_2ClF, ext. [D6]acetone) of $[Br(CH_2CF_3)_2][Sb(OTeF_5)_6]$ **1Br**. Signals of the external standard are marked with (*).

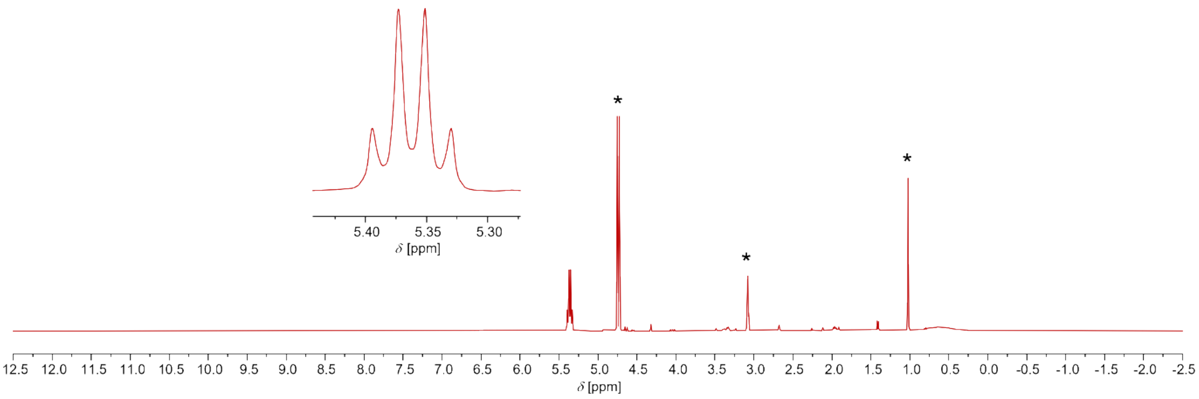

Figure S5. ^{1}H NMR spectrum (400 Hz, 18.8 °C, SO_2ClF, ext. [D6]acetone) of $[I(CH_2CF_3)_2][Sb(OTeF_5)_6]$ **1I**. Signals of the external standard are marked with (*).

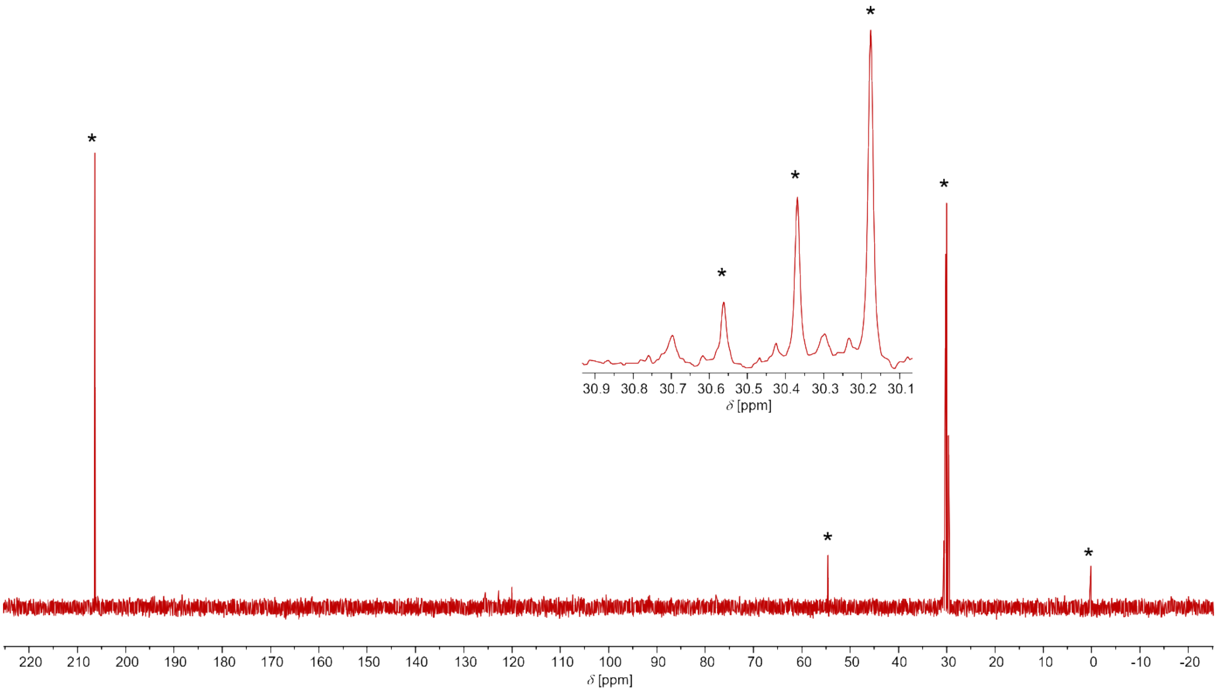

Figure S6. $^{13}C\{^{1}H\}$ NMR spectrum (101 Hz, 18 °C, SO_2ClF, ext. [D6]acetone) of $[I(CH_2CF_3)_2][Sb(OTeF_5)_6]$ **1I**. Signals of the external standard are marked with (*).

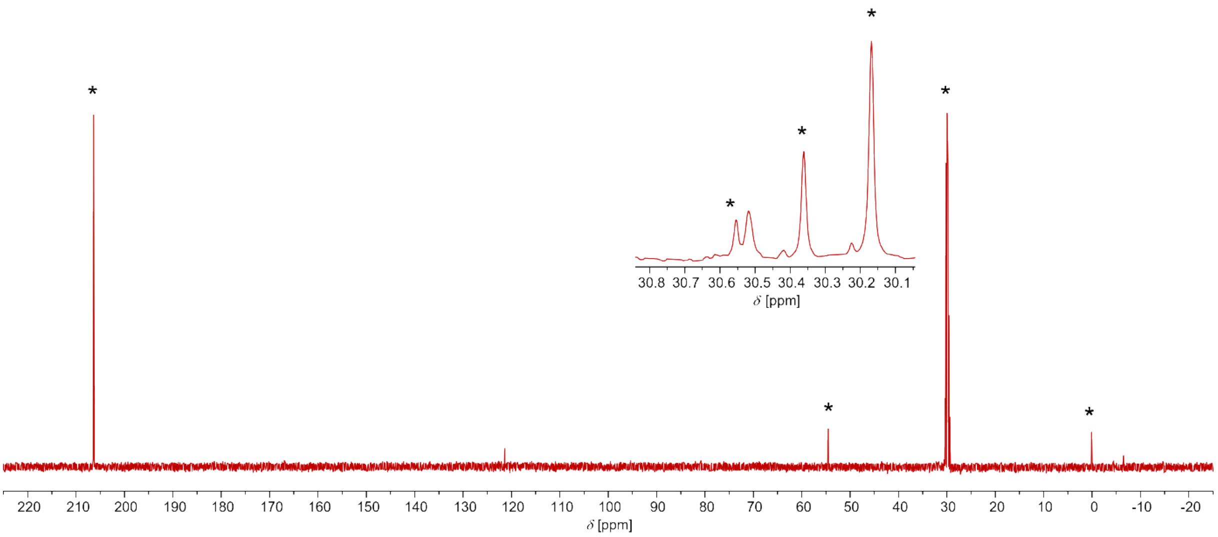

Figure S7. $^{13}C\{^{1}H,^{19}F\}$ NMR spectrum (101 Hz, 18.1 °C, SO_2ClF, ext. [D6]acetone) of $[I(CH_2CF_3)_2][Sb(OTeF_5)_6]$ **1I**. Signals of the external standard are marked with (*).

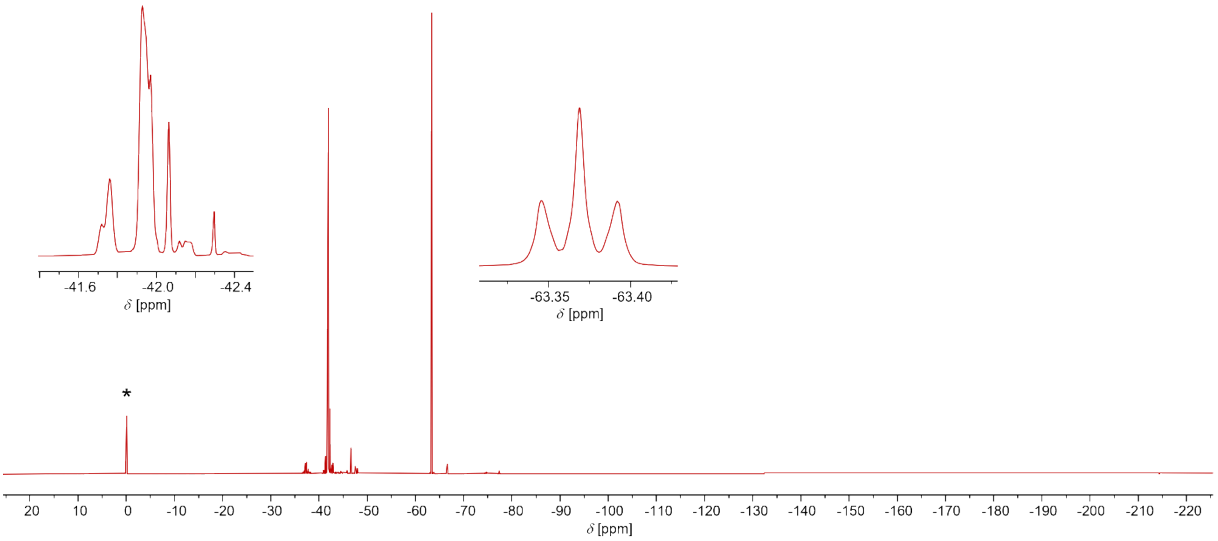

Figure S8. ^{19}F NMR spectrum (377 Hz, 18.7 °C, SO_2ClF, ext. [D6]acetone) of $[I(CH_2CF_3)_2][Sb(OTeF_5)_6]$ **1I**. Signals of the external standard are marked with (*).

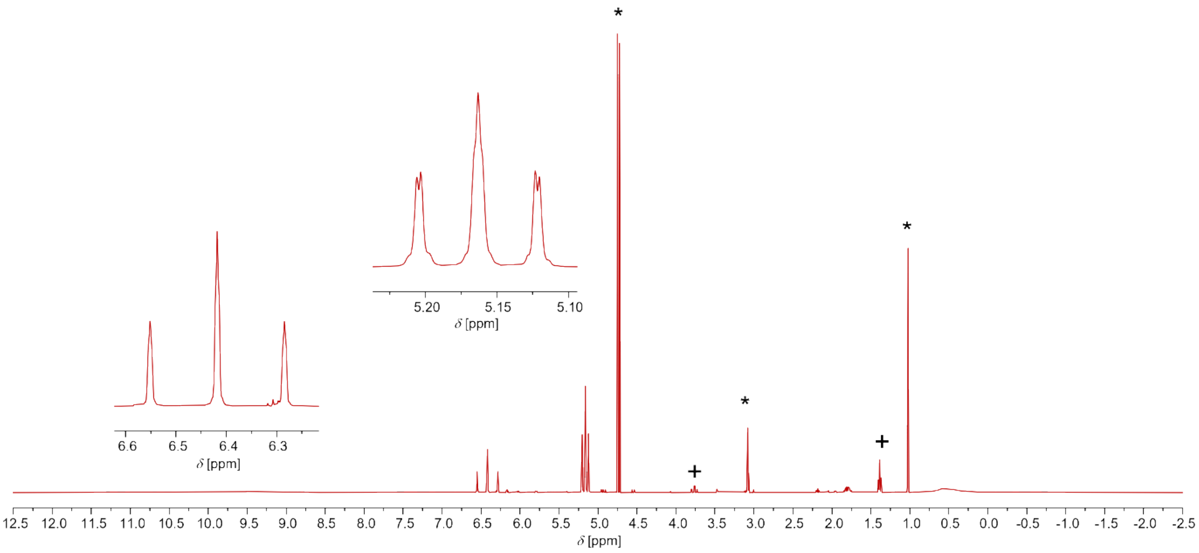

Figure S9. ^{1}H NMR spectrum (400 Hz, 19.1 °C, SO_2ClF, ext. [D6]acetone) of $[I(CH_2CHF_2)_2][Sb(OTeF_5)_6]$ **2**. Signals of the external standard are marked with (*). Signals of the starting material are marked with (+).

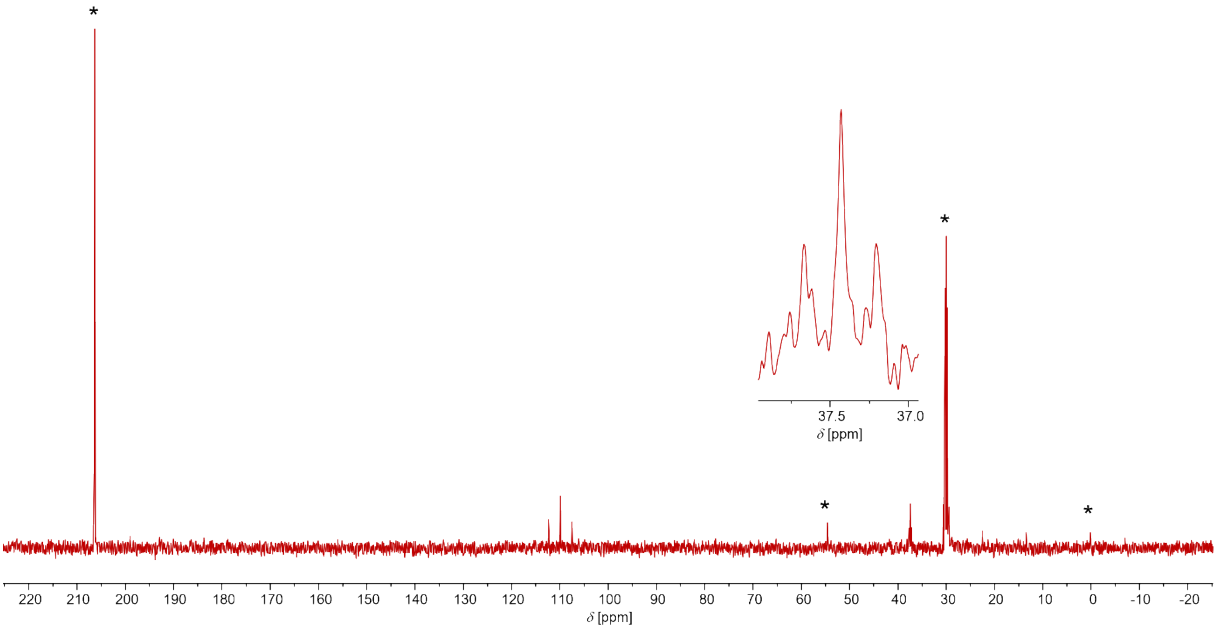

Figure S10. $^{13}C\{^{1}H\}$ NMR spectrum (101 Hz, 18.5 °C, SO_2ClF, ext. [D6]acetone) of $[I(CH_2CHF_2)_2][Sb(OTeF_5)_6]$ **2**. Signals of the external standard are marked with (*).

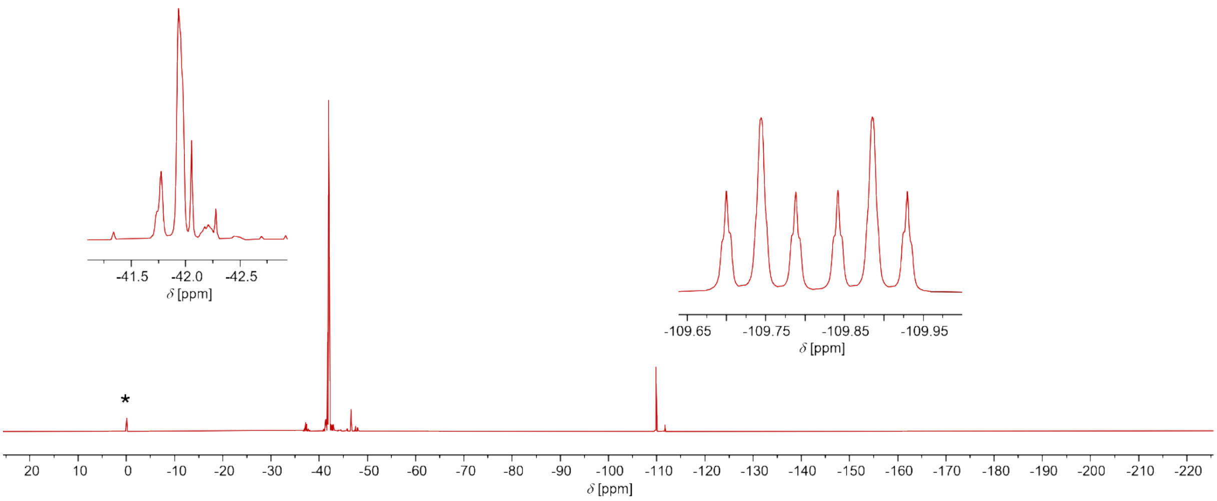

Figure S11. ^{19}F NMR spectrum (377 Hz, 19.1 °C, SO_2ClF, ext. [D6]acetone) of $[I(CH_2CHF_2)_2][Sb(OTeF_5)_6]$ **2**. Signals of the external standard are marked with (*).

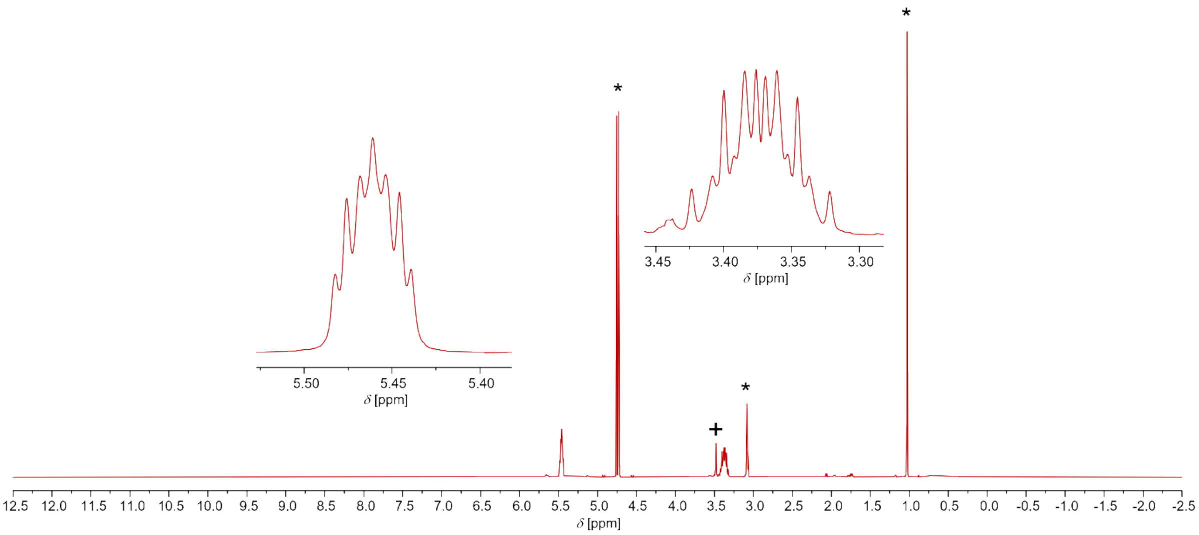

Figure S12. ^{1}H NMR spectrum (400 Hz, 20.5 °C, SO_2ClF, ext. [D6]acetone) of $[F_5C_5N(CH_2CH_2CF_3)][Sb(OTeF_5)_6]$ **4**. Signals of the external standard are marked with (*). Unidentified impurity is marked with (+).

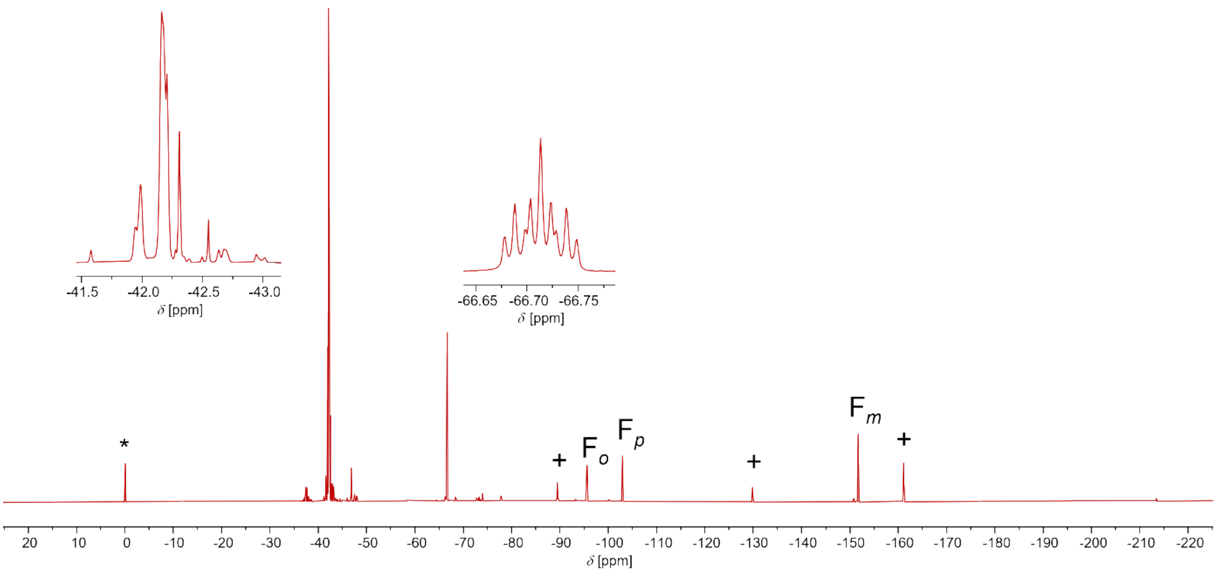

Figure S13. ^{19}F NMR spectrum (377 Hz, 20.5 °C, SO_2ClF, ext. [D6]acetone) of $[F_5C_5N(CH_2CH_2CF_3)][Sb(OTeF_5)_6]$ **4**. Signals of the external standard are marked with (*). Signals of the starting material NC_5F_5 are marked with (+).

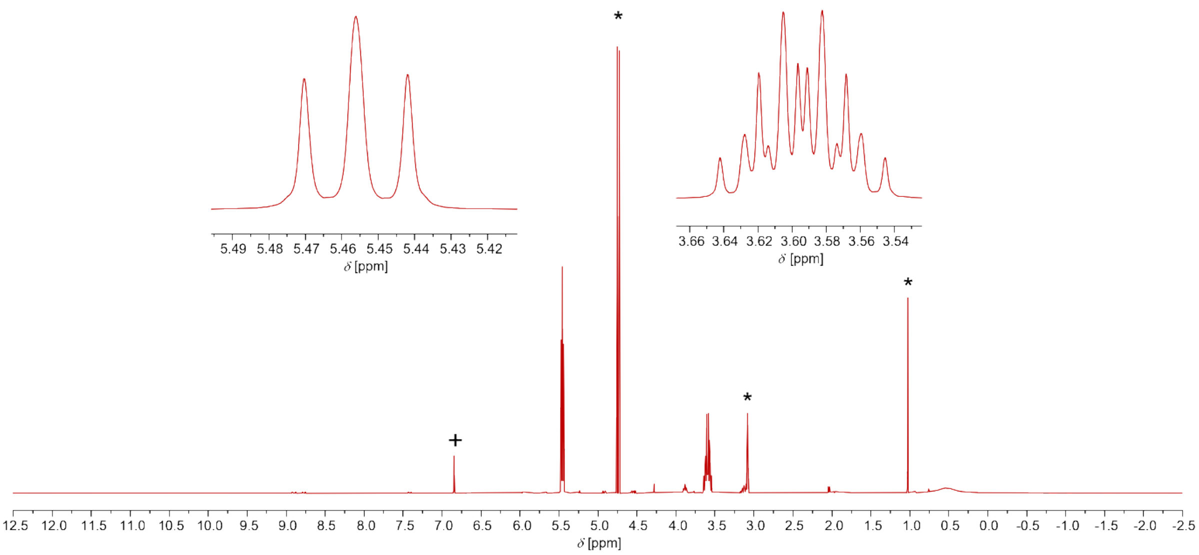

Figure S14. ^{1}H NMR spectrum (400 Hz, 19.1 °C, SO_2ClF, ext. [D6]acetone) of $[Br(CH_2CH_2CF_3)_2][Sb(OTeF_5)_6]$ **5Br**. Signals of the external standard are marked with (*). The signal of $HOTeF_5$ is marked with (+).

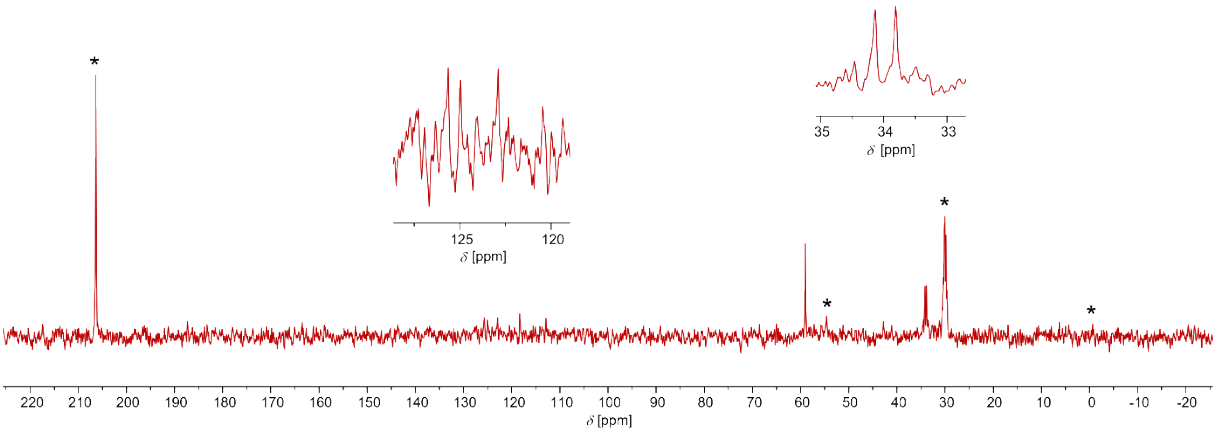

Figure S15. $^{13}C\{^{1}H\}$ NMR spectrum (101 Hz, 19.7 °C, SO_2ClF, ext. [D6]acetone) of $[Br(CH_2CH_2CF_3)_2][Sb(OTeF_5)_6]$ **5Br**. Signals of the external standard are marked with (*).

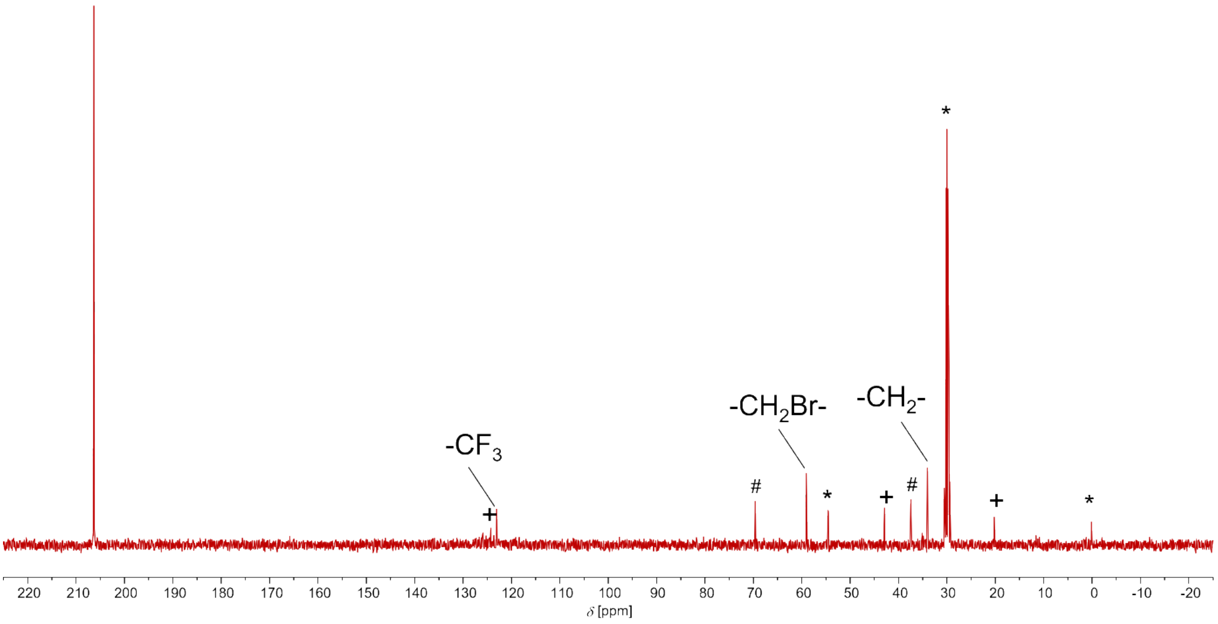

Figure S16. $^{13}C\{^{1}H,^{19}F\}$ NMR spectrum (101 Hz, 19.9 °C, SO_2ClF, ext. [D6]acetone) of $[Br(CH_2CH_2CF_3)_2][Sb(OTeF_5)_6]$ **5Br**. Signals of the external standard are marked with (*). Signals of residual starting material are marked with (+). Signals of unidentified decomposition products are marked with (#).

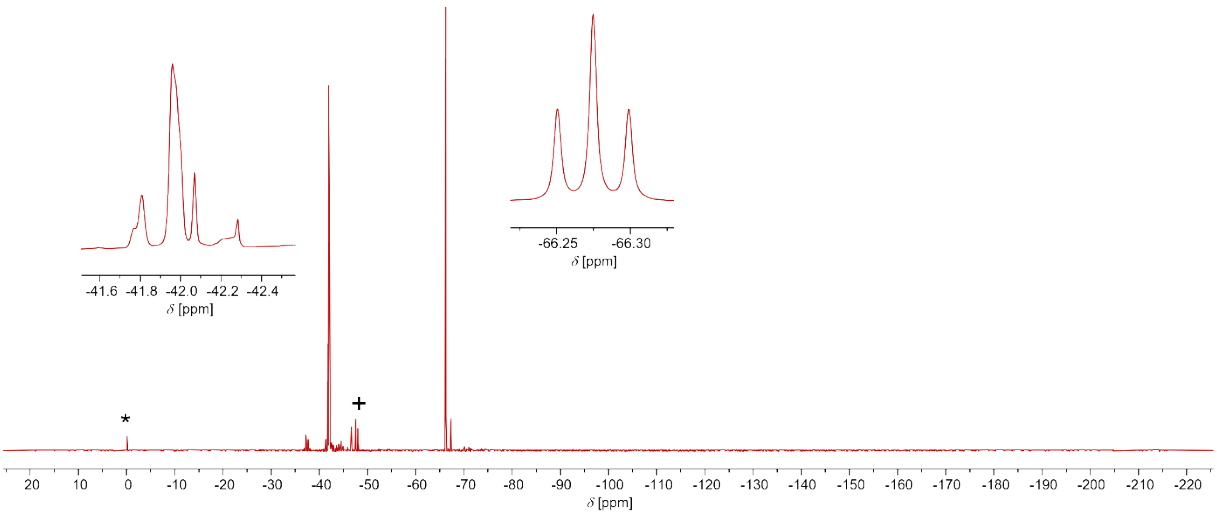

Figure S17. ^{19}F NMR spectrum (377 Hz, 19.1 °C, SO_2ClF, ext. [D6]acetone) of $[Br(CH_2CH_2CF_3)_2][Sb(OTeF_5)_6]$ **5Br**. Signals of the external standard are marked with (*). The signal of $HOTeF_5$ is marked with (+).

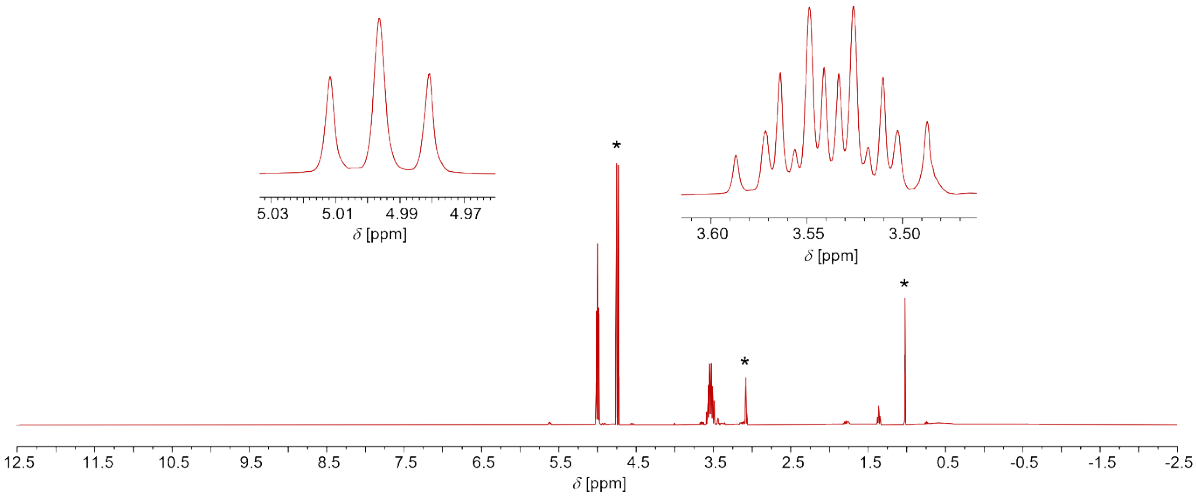

Figure S18. ^{1}H NMR spectrum (400 Hz, 18.7 °C, SO_2ClF, ext. [D6]acetone) of $[I(CH_2CH_2CF_3)_2][Sb(OTeF_5)_6]$ **5I**. Signals of the external standard are marked with (*).

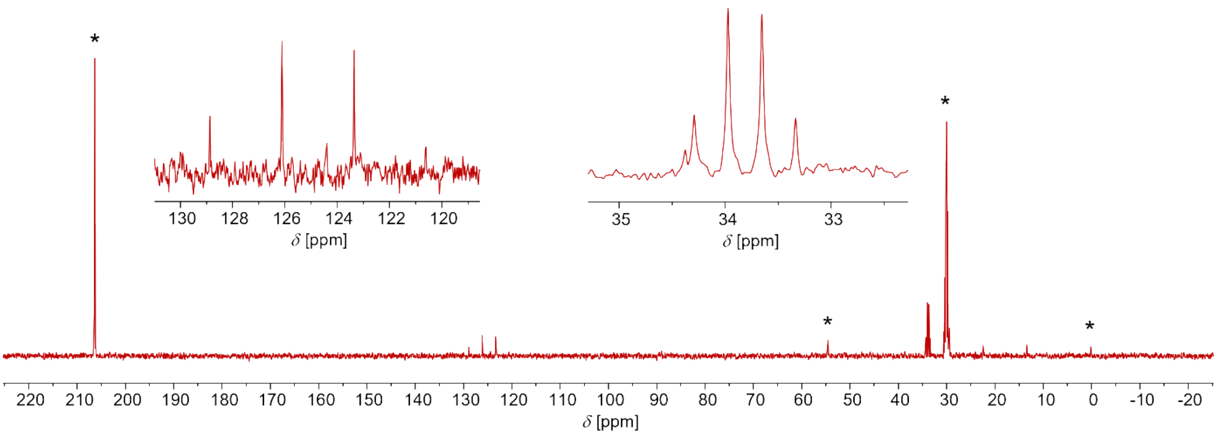

Figure S19. $^{13}C\{^{1}H\}$ NMR spectrum (101 Hz, 18.9 °C, SO_2ClF, ext. [D6]acetone) of $[I(CH_2CH_2CF_3)_2][Sb(OTeF_5)_6]$ **5I**. Signals of the external standard are marked with (*).

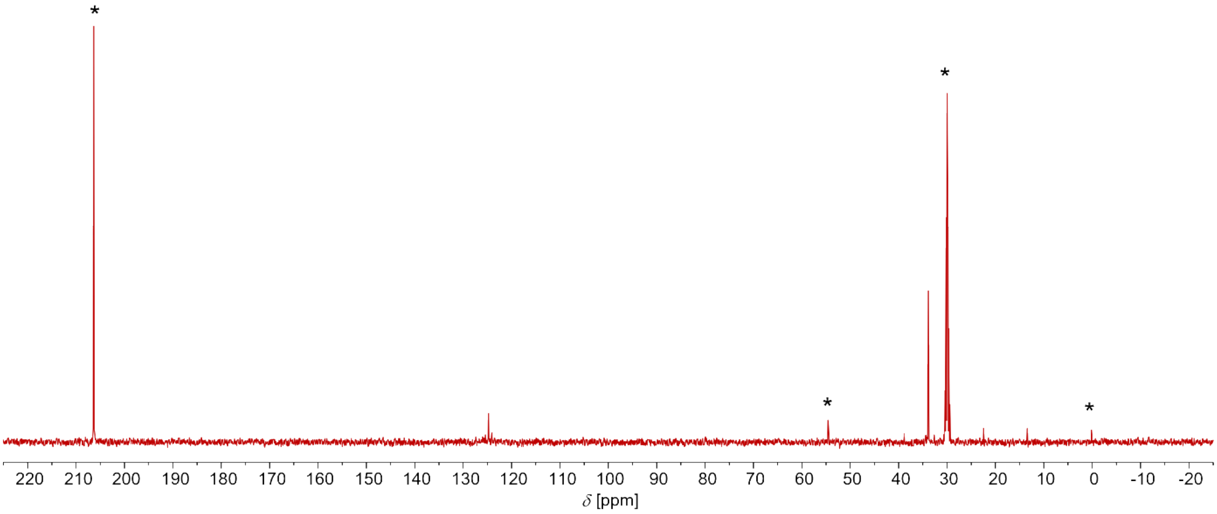

Figure S20. $^{13}C\{^{1}H,^{19}F\}$ NMR spectrum (101 Hz, 19 °C, SO_2ClF, ext. [D6]acetone) of $[I(CH_2CH_2CF_3)_2][Sb(OTeF_5)_6]$ **5I**. Signals of the external standard are marked with (*).

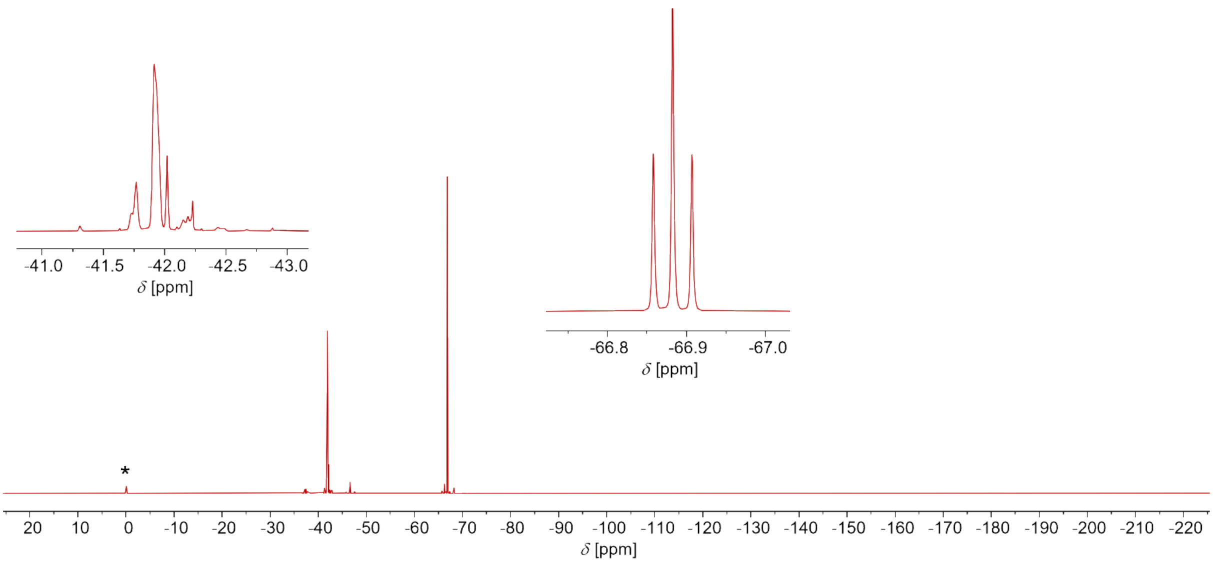

Figure S21. ^{19}F NMR spectrum (377 Hz, 18.8 °C, SO_2ClF, ext. [D6]acetone) of $[I(CH_2CH_2CF_3)_2][Sb(OTeF_5)_6]$ **5I**. Signals of the external standard are marked with (*).

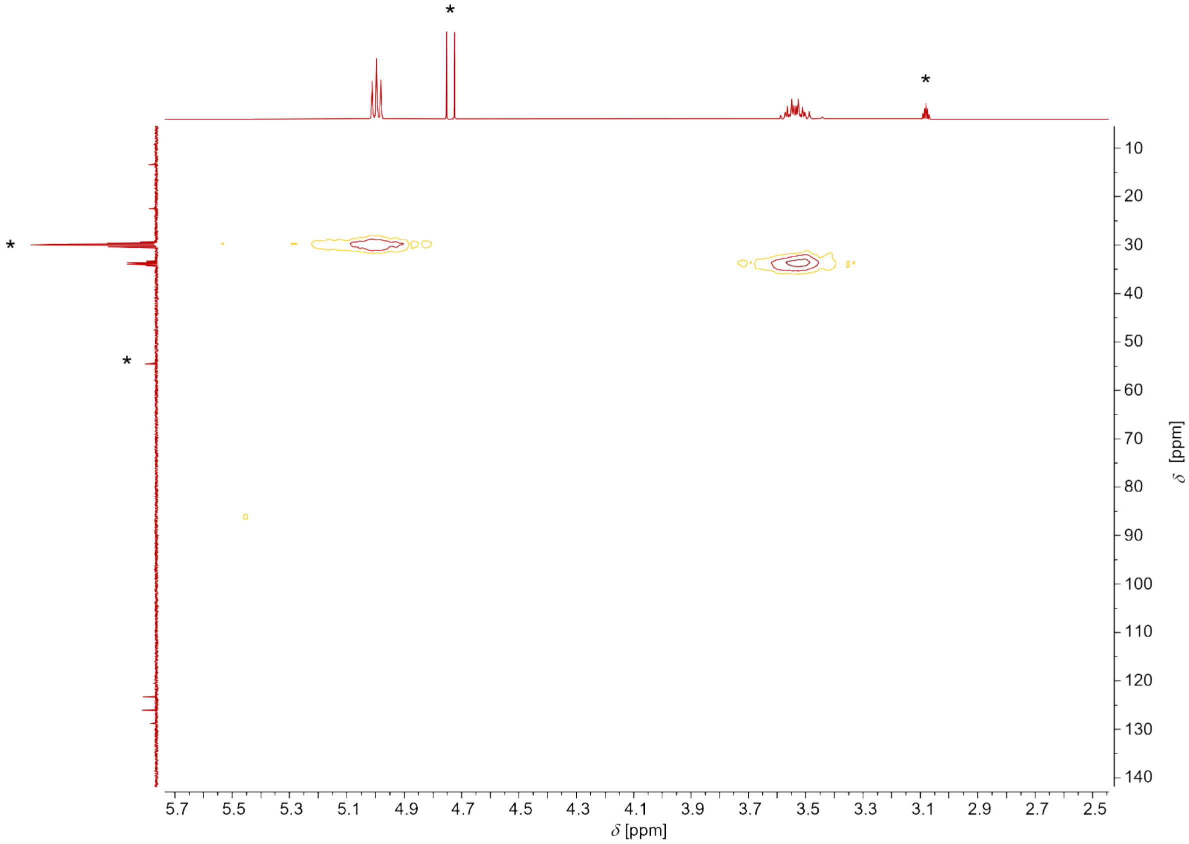

Figure S22. $^{1}H,^{13}C$-HMQC NMR spectrum (101 Hz, 18.9 °C, SO_2ClF, ext. [D6]acetone) of $[I(CH_2CH_2CF_3)_2][Sb(OTeF_5)_6]$ **5I**. Signals of the external standard are marked with (*).

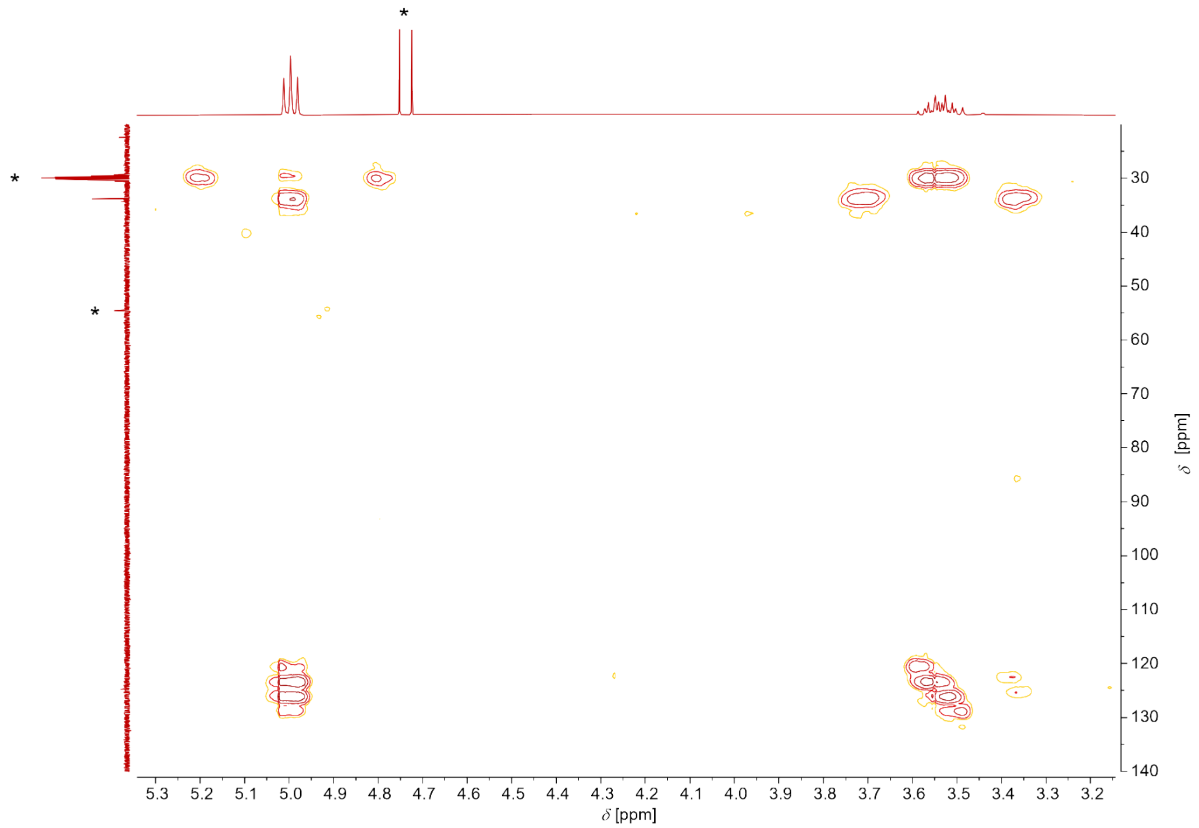

Figure S23. $^{1}H,^{13}C$-HMBC NMR spectrum (101 Hz, 18.7 °C, SO_2ClF, ext. [D6]acetone) of $[I(CH_2CH_2CF_3)_2][Sb(OTeF_5)_6]$ **5I**. Signals of the external standard are marked with (*).

3 IR spectra

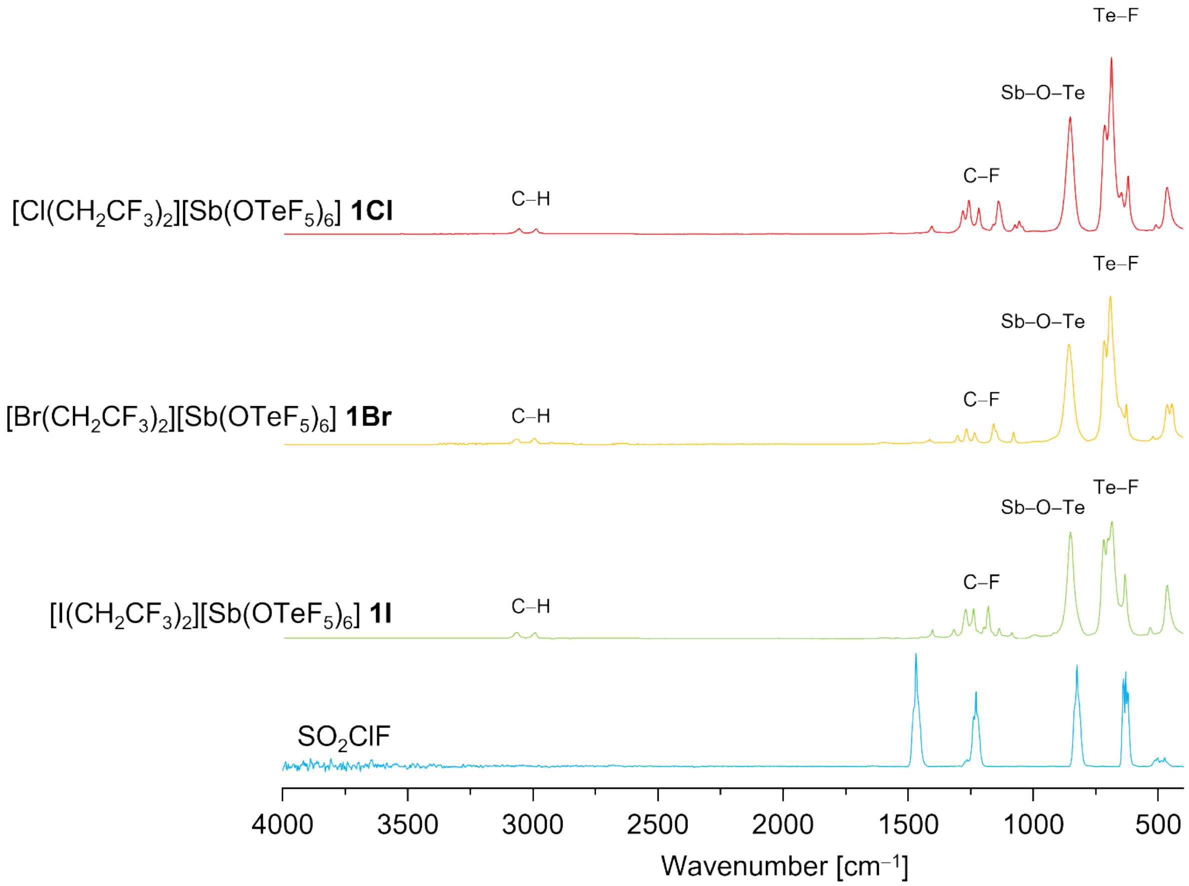

Figure S24. IR spectra (ATR, 20 °C) of $[Cl(CH_2CF_3)_2][Sb(OTeF_5)_6]$ **1Cl**, $[Cl(CH_2CF_3)_2][Sb(OTeF_5)_6]$ **1Br**, $[I(CH_2CF_3)_2][Sb(OTeF_5)_6]$ **1I** and SO_2ClF. The spectrum of SO_2ClF was recorded in the gas phase.

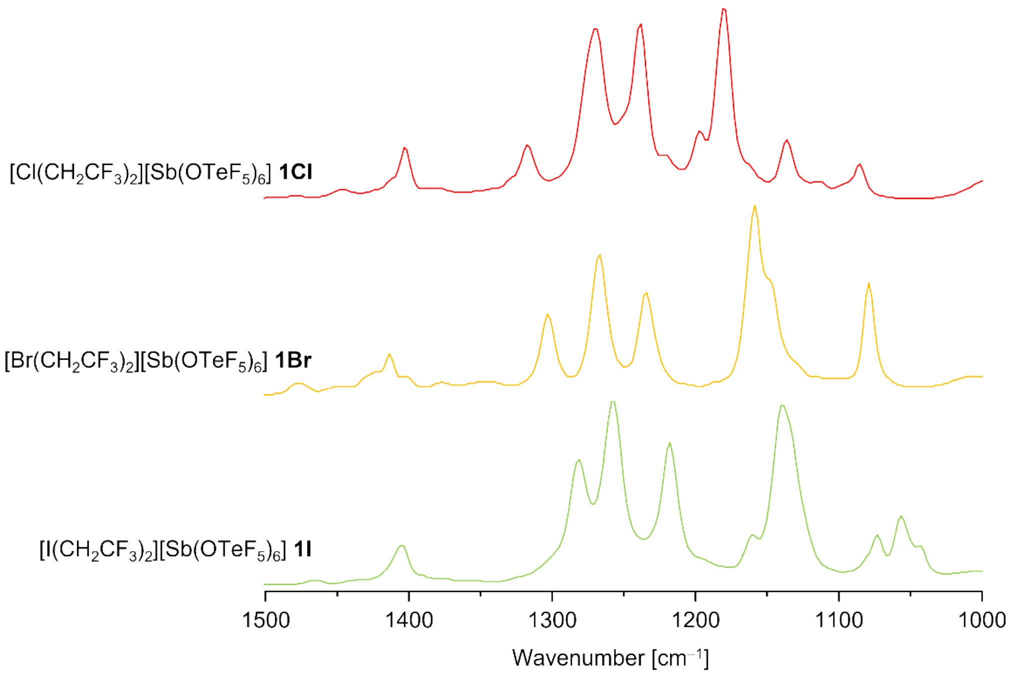

Figure S25. Zoom into the IR spectra in the range of 1000 to 1500 cm^{-1} (ATR, 20 °C) of $[Cl(CH_2CF_3)_2][Sb(OTeF_5)_6]$ **1Cl**, $[Cl(CH_2CF_3)_2][Sb(OTeF_5)_6]$ **1Br** and $[I(CH_2CF_3)_2][Sb(OTeF_5)_6]$ **1I**.

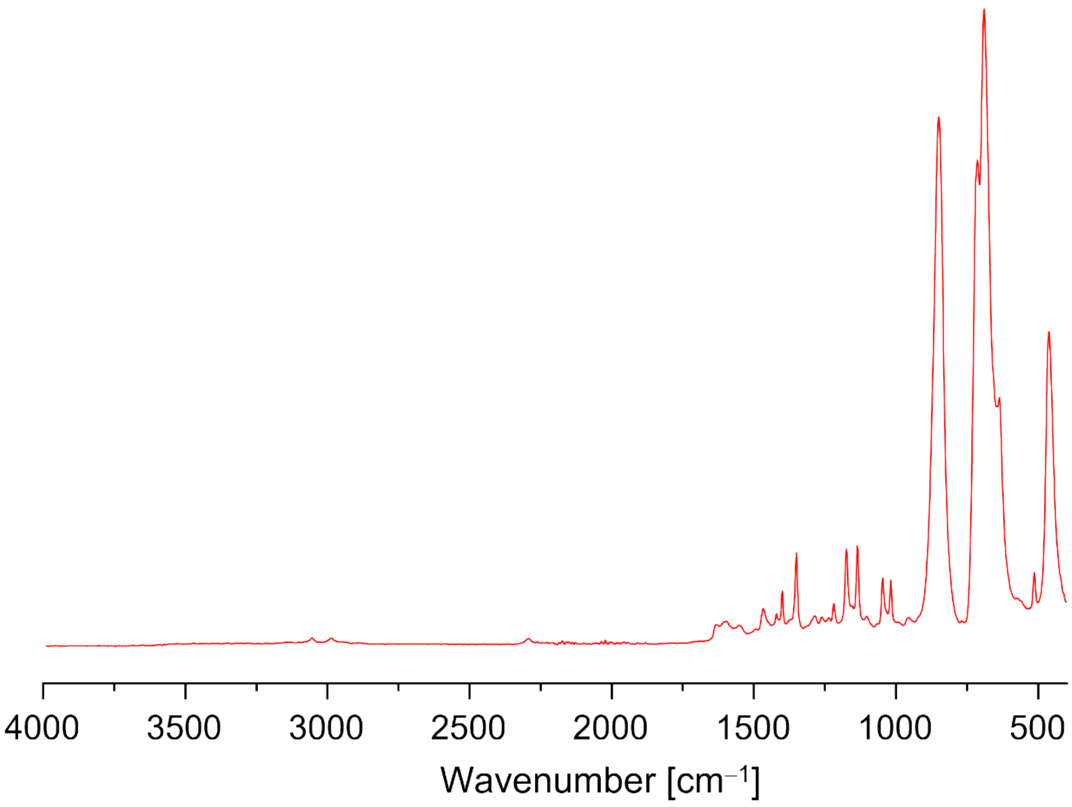

Figure S26. IR spectrum (ATR, 20 °C) of $[I(CH_2CHF_2)_2][Sb(OTeF_5)_6]$ (**2**).

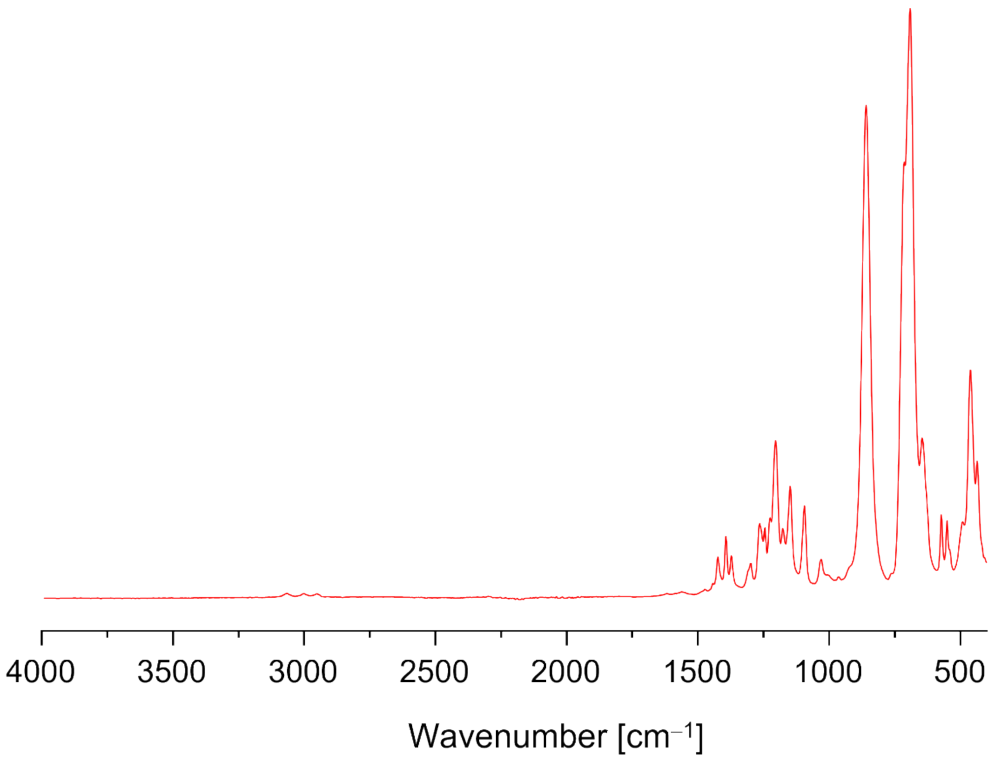

Figure S27. IR spectrum (ATR, 20 °C) of $[Br(CH_2CH_2CF_3)_2][Sb(OTeF_5)_6]$ (**5Br**).

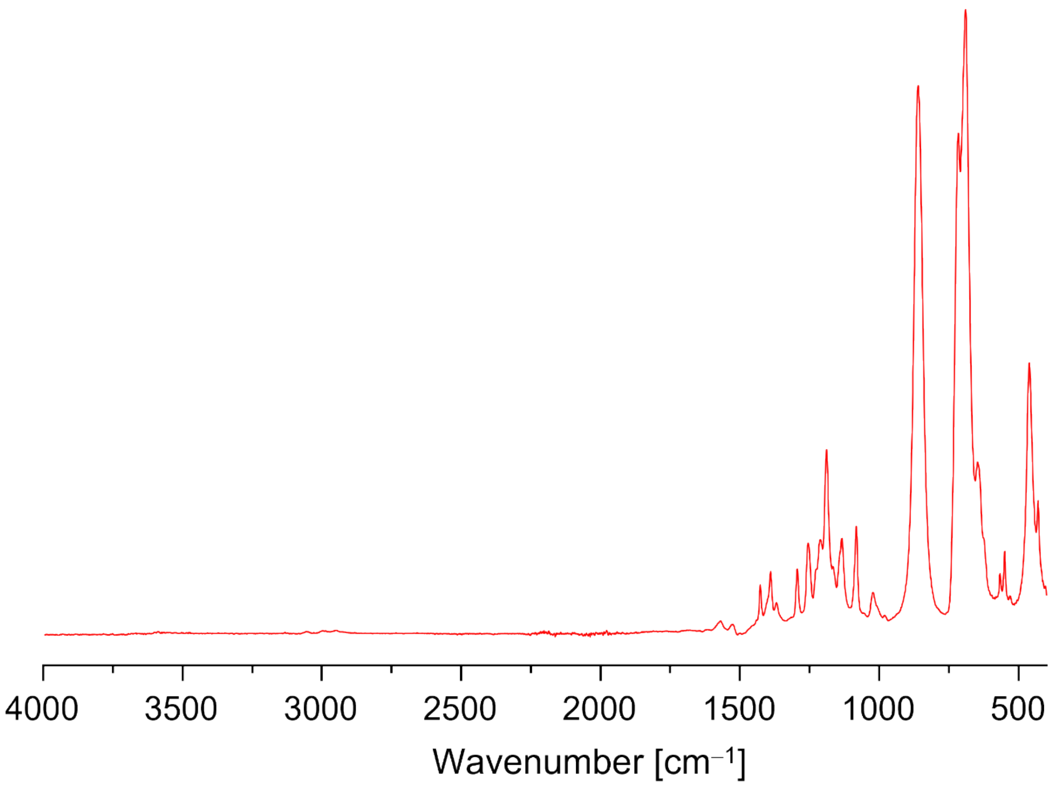

Figure S28. IR spectrum (ATR, 20 °C) of $[I(CH_2CH_2CF_3)_2][Sb(OTeF_5)_6]$ (**5I**).

4 Raman spectra

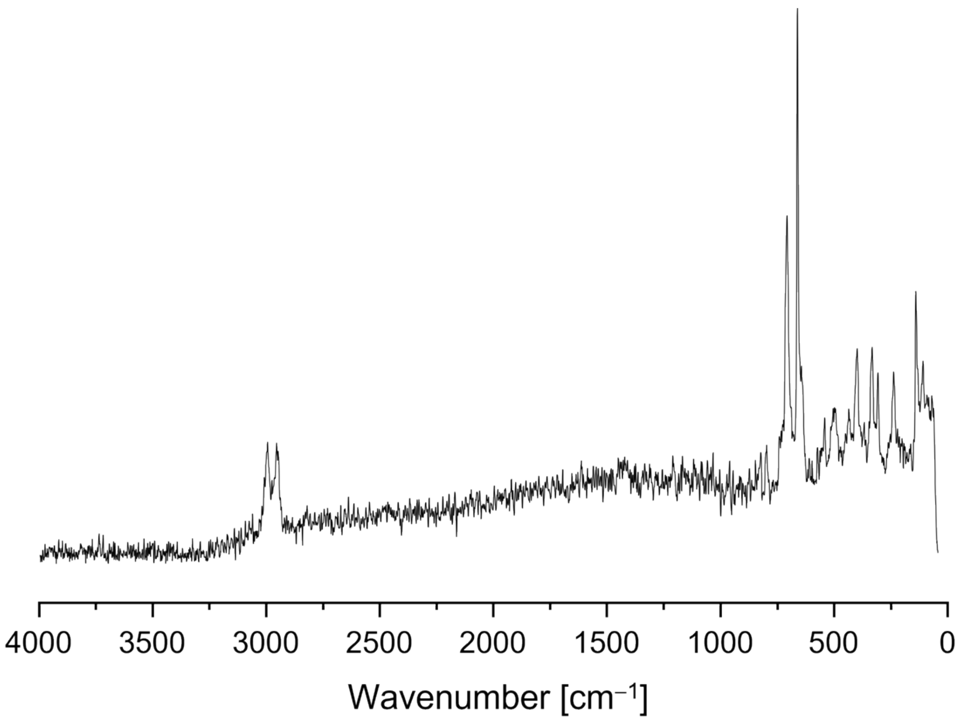

Figure S29. IR spectrum (20 °C) of $[Br(CH_2CH_2CF_3)_2][Sb(OTeF_5)_6]$ (**5Br**).

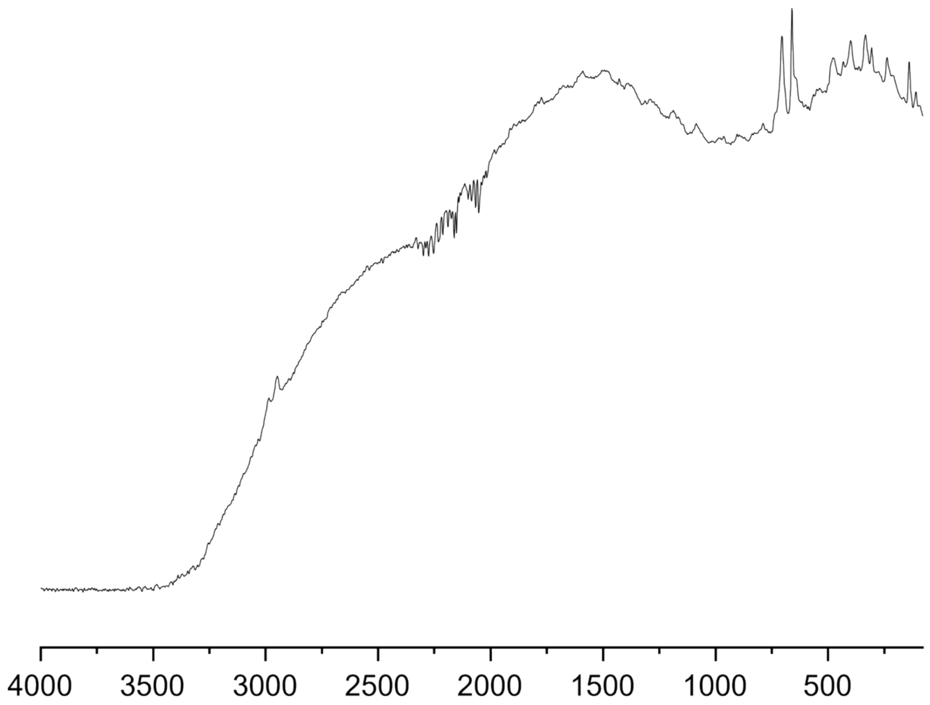

Figure S30. IR spectrum (20 °C) of $[I(CH_2CH_2CF_3)_2][Sb(OTeF_5)_6]$ (**5I**).

5 Crystal data

Table S1. Crystallographic details.

	**[Cl(CH$_2$CF$_3$)$_2$][Sb(OTeF$_5$)$_6$]·3 SO$_2$ClF (1Cl)**	**[Br(CH$_2$CF$_3$)$_2$][Sb(OTeF$_5$)$_6$]·3 SO$_2$ClF (1Br)**	**[I(CH$_2$CHF$_2$)$_2$][Sb(OTeF$_5$)$_6$] (2I)**	**[C$_4$H$_9$][Sb(OTeF$_5$)$_6$] (3)**
CCDC number	2453012	2453010	2453013	2453011
Empirical formula	$C_4H_4Cl_4F_{39}O_{12}S_3SbTe_6$	$C_4H_4BrCl_3F_{39}O_{12}S_3SbTe_6$	$C_4H_6F_{34}IO_6SbTe_6$	$C_4H_9F_{30}O_6SbTe_6$
Formula weight	2110.40	2154.86	1810.34	1610.46
Temperature [K]	100(2)	100(2)	100(2)	100(2)
Crystal system	triclinic	triclinic	triclinic	trigonal
Space group (number)	$P\bar{1}$ (2)	$P\bar{1}$ (2)	$P\bar{1}$ (2)	$R3$ (146)
a [Å]	9.8884(8)	9.8994(7)	9.9568(7)	13.8891(10)
b [Å]	10.0411(8)	10.0636(7)	10.0522(6)	13.8891(10)
c [Å]	22.687(2)	22.7766(17)	10.7376(8)	13.2594(14)
α [°]	93.500(4)	93.244(3)	92.260(3)	90
β [°]	100.671(3)	100.968(3)	114.527(3)	90
γ [°]	95.798(3)	95.729(3)	119.573(2)	120
Volume [Å^3]	2195.0(3)	2209.9(3)	808.81(10)	2215.1(4)
Z	2	2	1	3
ρ_{calc} [gcm^{-3}]	3.193	3.238	3.717	3.622
μ [mm^{-1}]	5.133	5.942	7.333	6.957
F(000)	1908	1944	800	2142
Crystal size [mm^3]	0.402×0.457×0.792	0.357×0.434×0.479	0.051×0.121×0.125	0.081×0.169×0.185
Crystal colour	colourless	colourless	colourless	colourless
Crystal shape	block	block	plate	block
Radiation	MoK_α (λ=0.71073 Å)	MoK_α (λ=0.71073 Å)	MoK_α (λ=0.71073 Å)	MoK_α (λ=0.71073 Å)
2θ range [°]	4.22 to 56.85 (0.75 Å)	4.08 to 61.42 (0.70 Å)	4.38 to 56.68 (0.75 Å)	4.57 to 61.12 (0.70 Å)
Index ranges	−13 ≤ h ≤ 13<br>−13 ≤ k ≤ 13<br>−30 ≤ l ≤ 30	−14 ≤ h ≤ 14<br>−14 ≤ k ≤ 14<br>−32 ≤ l ≤ 32	−13 ≤ h ≤ 11<br>−13 ≤ k ≤ 13<br>−14 ≤ l ≤ 14	−19 ≤ h ≤ 19<br>−19 ≤ k ≤ 19<br>−18 ≤ l ≤ 18
Reflections collected	63088	156080	4035	84459
Independent reflections	10988<br>R_{int} = 0.0773<br>R_{sigma} = 0.0507	13677<br>R_{int} = 0.0530<br>R_{sigma} = 0.0246	4035<br>R_{int} = 0.0719<br>R_{sigma} = 0.0322	3014<br>R_{int} = 0.0767<br>R_{sigma} = 0.0215
Completeness to θ = 25.242°	99.7 %	100.0 %	99.9 %	99.9 %
Data / Restraints / Parameters	10988 / 0 / 625	13677 / 0 / 625	4035 / 48 / 278	3014 / 49 / 144
Absorption correction T_{min}/T_{max} (method)	0.0873 / 1.0000<br>(numerical)	0.4236 / 0.7461<br>(multi-scan)	0.5260 / 0.7457<br>(multi-scan)	0.5831 / 0.7461<br>(multi-scan)
Goodness-of-fit on F^2	1.187	1.137	1.085	1.078
Final *R* indexes [I≥2σ(I)]	R_1 = 0.0368<br>wR_2 = 0.0879	R_1 = 0.0289<br>wR_2 = 0.0549	R_1 = 0.0316<br>wR_2 = 0.0667	R_1 = 0.0278<br>wR_2 = 0.0524
Final *R* indexes [all data]	R_1 = 0.0404<br>wR_2 = 0.0899	R_1 = 0.0364<br>wR_2 = 0.0569	R_1 = 0.0342<br>wR_2 = 0.0685	R_1 = 0.0338<br>wR_2 = 0.0556
Largest peak/hole [eÅ^{-3}]	1.30/−2.12	1.04/−1.02	0.98/−1.09	1.60/−1.04

6 Quantum-chemical calculations

All calculations have been performed using Gaussian 16.[10] The hybrid functional B3LYP[11] has been used in conjunction with the cc-pVTZ basis set[12] (and D3[13]). Minima on potential energy surfaces were characterised by normal mode analysis. For visualization, the program ChemCraft v1.8 was used.[14]

6.1 QTAIM analysis

Table S2. QTAIM analysis of **cat1** in the gas phase.

	ρ_{BCP} [Å^{-3}]	$\nabla^2\rho_{BCP}$ [Å^{-5}]	ELF_{BCP}	$\frac{\lvert V_{BCP}\rvert}{G_{BCP}}$	ε_{BCP}
C1–C2	2.22	–26.20	0.93	4.24	0.21
H–$\pi_{C1\text{-}C2}$	1.24	–5.86	0.88	2.97	1.78
C2–C3	1.77	–17.12	0.98	5.63	0.01

Table S3. QTAIM analysis of **cat2** with a solvent model applied (ε=100).

	ρ_{BCP} [Å^{-3}]	$\nabla^2\rho_{BCP}$ [Å^{-5}]	ELF_{BCP}	$\frac{\lvert V_{BCP}\rvert}{G_{BCP}}$	ε_{BCP}
C1–C2	2.22	–26.56	0.94	4.32	0.19
H–C2	1.34	–10.94	0.93	4.19	0.49
C2–C3	1.79	–17.74	0.98	5.64	0.01

6.2 Optimized structures

$CH_2ClCH_2CF_3$ (C_1)

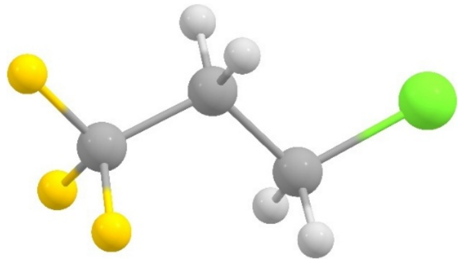

In the gas phase

Cl	0.77559	1.50206	-0.78941
C	-0.72966	1.10792	0.12507
H	-0.41772	0.72134	1.08969
H	-1.26425	2.04161	0.26533
C	-1.55856	0.09527	-0.64556
H	-1.83716	0.49039	-1.62111
H	-0.99483	-0.82313	-0.80107
C	-2.83179	-0.26638	0.08300
F	-2.58223	-0.80576	1.29553
F	-3.61928	0.81163	0.28606
F	-3.55254	-1.16328	-0.61360

With solvent model

Cl	-2.777906	0.143415	-0.000025
C	-1.102527	-0.543897	0.000019
H	-1.026080	-1.163020	0.886921
H	-1.026097	-1.163172	-0.886777
C	-0.082728	0.581312	-0.000088
H	-0.198944	1.207887	-0.882523
H	-0.198981	1.208086	0.882202
C	1.332227	0.057799	0.000006
F	1.597059	-0.704886	1.084472
F	1.597085	-0.705208	-1.084231
Cl	2.227264	1.065747	-0.000133

$CHCl(CH_3)(CF_3)$ (C_1)

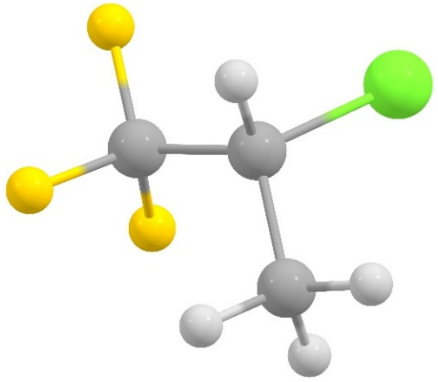

In the gas phase

Cl	1.81971	-0.61528	0.01845
C	0.45080	0.45033	-0.48220
H	0.43329	0.42398	-1.56851
C	-0.85939	-0.16962	-0.00905
C	0.64730	1.85956	0.04004
H	1.57865	2.26704	-0.34513
H	-0.17540	2.49433	-0.28799
H	0.68598	1.86446	1.12716
F	-0.95015	-0.22429	1.32779
F	-1.03657	-1.40791	-0.48746
F	-1.88993	0.58424	-0.45497

With solvent model

Cl	1.814207	-0.621326	0.018728
C	0.449709	0.461037	-0.485625
H	0.438901	0.437204	-1.571133
C	-0.856380	-0.163377	-0.012791
C	0.652313	1.865785	0.042326
H	1.583827	2.269854	-0.345057
H	-0.165856	2.502770	-0.291763
H	0.687445	1.873186	1.129499
F	-0.941303	-0.237789	1.326843
F	-1.036325	-1.403062	-0.498528
F	-1.895670	0.585169	-0.439803

$[CH_2CH_2CF_3]^+$ (C_1)

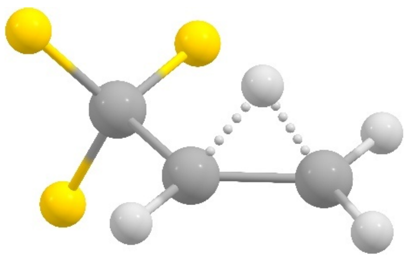

In the gas phase

C	-0.92842	-0.64943	0.01708
H	-0.94168	-1.73632	0.02341
H	-1.61944	-0.27201	-1.08873
C	0.46370	0.00383	0.00129
C	-2.09634	0.07666	0.06252
H	-3.05387	-0.42863	0.13518
H	-2.06613	1.16164	0.08224
F	1.17946	-0.51073	-0.98697
F	0.37014	1.32014	-0.14682
F	1.01124	-0.28841	1.17408

With solvent model

C	-0.94555	-0.64624	-0.00909
H	-0.96856	-1.71818	0.15300
H	-1.45965	-0.48461	-1.11372
C	0.44011	-0.00512	0.00029
C	-2.10194	0.09634	0.06943
H	-3.05963	-0.40577	0.13445
H	-2.07707	1.17534	-0.03019
F	1.23805	-0.61291	-0.87694
F	0.38877	1.29538	-0.29260
F	0.95199	-0.15321	1.22429

[Cl(CH$_2$CH$_2$CF$_3$)$_2$]$^+$ (C_1)

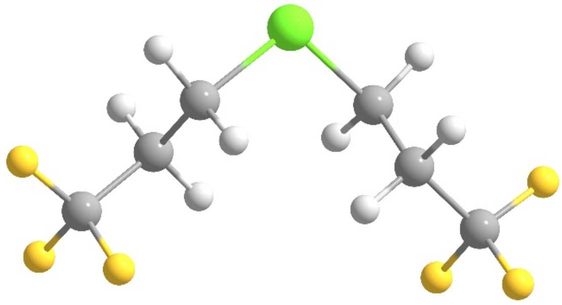

In the gas phase

Cl	0.83049	1.53907	-0.80119
C	-0.75770	1.08442	0.12685
H	-0.40415	0.71838	1.08325
H	-1.22391	2.05942	0.22302
C	-1.54527	0.08260	-0.67940
H	-1.83604	0.48611	-1.64729
H	-0.99199	-0.84151	-0.82550
C	-2.82410	-0.26228	0.08437
F	-2.52650	-0.78256	1.28952
F	-3.56868	0.83817	0.28731
F	-3.54489	-1.14727	-0.59538
C	2.31032	0.67329	0.03926
H	3.08705	1.00194	-0.64834
C	2.48841	1.16247	1.44449
H	1.66894	0.86854	2.09606
H	3.39861	0.69638	1.82981
H	2.62428	2.24035	1.47219
C	2.11033	-0.84068	-0.11941
F	1.09986	-1.27655	0.65122
F	1.84635	-1.17355	-1.38238
F	3.23463	-1.43521	0.26751

With solvent model

Cl	-0.000008	2.018134	0.000073
C	1.353494	0.898981	0.607347
H	1.948808	1.602039	1.180331
H	0.854501	0.196021	1.263258
C	2.083783	0.276928	-0.560068
H	1.435581	-0.370386	-1.145566
H	2.501148	1.040684	-1.211638
C	3.232122	-0.580569	-0.060633
F	4.122657	0.138799	0.649861
F	2.801930	-1.579065	0.735968
F	3.888134	-1.136940	-1.089753
C	-1.353820	0.899405	-0.607318
H	-0.855161	0.196940	-1.263995
C	-1.949461	1.602829	-1.179506
H	-2.083421	0.276469	0.560053
H	-2.500396	1.039696	1.212490
H	-1.434834	-0.371298	1.144634
C	-3.232050	-0.580641	0.060601
F	-4.123110	0.139330	-0.648632
F	-3.887347	-1.138001	1.089642
F	-2.802342	-1.578373	-0.737213

[Cl(CH$_2$CH$_2$CF$_3$)(CH(CH$_3$)(CF$_3$))]$^+$ (C_1)

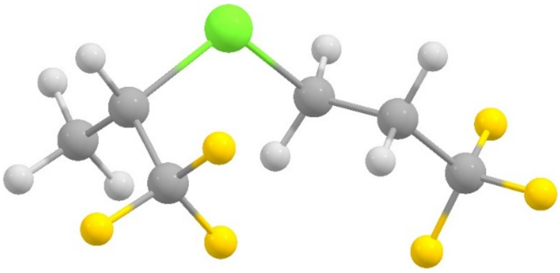

In the gas phase

Cl	0.83049	1.53907	-0.80119
C	-0.75770	1.08442	0.12685
H	-0.40415	0.71838	1.08325
H	-1.22391	2.05942	0.22302
C	-1.54527	0.08260	-0.67940
H	-1.83604	0.48611	-1.64729
H	-0.99199	-0.84151	-0.82550
C	-2.82410	-0.26228	0.08437
F	-2.52650	-0.78256	1.28952
F	-3.56868	0.83817	0.28731
F	-3.54489	-1.14727	-0.59538
C	2.31032	0.67329	0.03926
H	3.08705	1.00194	-0.64834
C	2.48841	1.16247	1.44449
H	1.66894	0.86854	2.09606
H	3.39861	0.69638	1.82981
H	2.62428	2.24035	1.47219
C	2.11033	-0.84068	-0.11941
F	1.09986	-1.27655	0.65122
F	1.84635	-1.17355	-1.38238
F	3.23463	-1.43521	0.26751

With solvent model

Cl	0.776619	1.433326	-0.871793
C	-0.778072	1.042292	0.096749
H	-0.419128	0.712433	1.062894
H	-1.240036	2.019916	0.164287
C	-1.585240	0.013877	-0.660823
H	-1.848518	0.372132	-1.653521
H	-1.046981	-0.925473	-0.753041
C	-2.877918	-0.275752	0.079826
F	-2.646476	-0.748495	1.321042
F	-3.640899	0.826874	0.211054
F	-3.601782	-1.194436	-0.576248
C	2.266829	0.744975	0.074297
H	3.052105	1.164811	-0.548719
C	2.296976	1.238531	1.489325
H	1.500184	0.821124	2.098627
H	3.248154	0.913746	1.913725
H	2.264754	2.323361	1.518197
C	2.248137	-0.776575	-0.093173
F	1.189442	-1.326484	0.526490
F	2.212949	-1.142005	-1.375696
F	3.360396	-1.270194	0.460116

[Cl(CH(CH$_3$)(CF$_3$))$_2$]$^+$ (C_1)

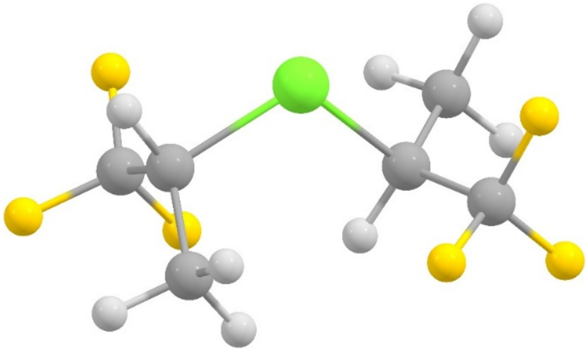

In the gas phase

Cl	-0.00695	0.17012	-1.19389
C	1.52192	-0.80111	-0.58608
H	1.97406	-0.98772	-1.55831
C	-1.11090	0.62759	0.31432
H	-0.50625	0.31280	1.15691
C	2.39475	0.19759	0.18799
C	1.13200	-2.04431	0.15243
H	0.50276	-2.68471	-0.45898
H	2.05804	-2.58205	0.36984
H	0.63959	-1.83376	1.09804
C	-1.36795	2.10276	0.25099
H	-2.00715	2.34879	1.10252
H	-0.44302	2.66507	0.34711
H	-1.89133	2.38883	-0.65870
C	-2.34773	-0.27140	0.18133
F	1.79741	0.57447	1.33352
F	2.64619	1.28964	-0.52977
F	3.53987	-0.41068	0.48163
F	-2.00726	-1.56585	0.20114
F	-3.13544	-0.01859	1.22589
F	-3.01200	-0.02298	-0.94665

With solvent model

Cl	0.005115	-0.203204	-1.170295
C	-1.490470	0.805547	-0.565001
H	-1.915217	1.045570	-1.535963
C	1.106069	-0.603850	0.329898
H	0.523518	-0.246125	1.170546
C	-2.416579	-0.173500	0.164346
C	-1.072527	2.008284	0.222197
H	-0.413819	2.642357	-0.361933
H	-1.984156	2.569302	0.436023
H	-0.608043	1.753158	1.170291
C	1.352866	-2.084116	0.344976
H	1.988948	-2.289076	1.207567
H	0.421917	-2.627807	0.472060
H	1.864313	-2.421949	-0.552391
C	2.358799	0.262604	0.157015
F	-1.881142	-0.590058	1.323938
F	-2.695795	-1.248236	-0.574001
F	-3.558057	0.466796	0.435373
F	2.061668	1.566361	0.106360
F	3.152138	0.052757	1.213522
F	3.033037	-0.054373	-0.953167

$[F_5C_5N(CH_2CH_2CF_3)]^+$ (C_1)

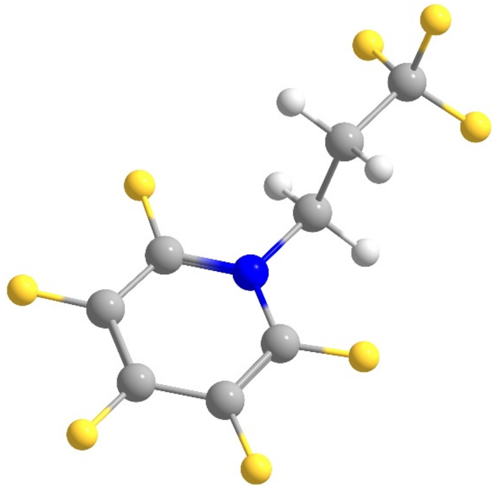

In the gas phase

C	-0.941175	-1.167820	0.245470
C	-2.290889	-1.205752	-0.039364
C	-2.978167	-0.000010	-0.183573
C	-2.290896	1.205744	-0.039365
C	-0.941188	1.167833	0.245467
F	-0.249153	2.263584	0.382476
F	-2.906232	2.355402	-0.173933
F	-4.250008	-0.000007	-0.455271
F	-2.906198	-2.355424	-0.173927
F	-0.249140	-2.263571	0.382480
N	-0.276370	0.000005	0.395280
C	2.018271	0.000031	-0.576060
H	1.815567	-0.885990	-1.175793
H	1.815599	0.886085	-1.175754
C	1.192701	0.000016	0.706583
H	1.383763	-0.881831	1.308120
H	1.383747	0.881859	1.308130
C	3.501883	-0.000004	-0.239951
F	4.235444	0.000041	-1.350875
F	3.824470	1.085925	0.486412
F	3.824451	-1.085992	0.486316

With solvent model

C	-0.943739	-1.167317	0.247318
C	-2.290008	-1.201135	-0.041368
C	-2.972440	0.001747	-0.187436
C	-2.287024	1.202892	-0.039942
C	-0.941015	1.164834	0.248660
F	-0.249708	2.260169	0.389988
F	-2.904449	2.357466	-0.175097
F	-4.249137	0.003457	-0.463441
F	-2.910378	-2.354019	-0.177730
F	-0.256899	-2.265472	0.387561
N	-0.279664	-0.002093	0.398505
C	2.010656	0.030108	-0.553337
H	1.791597	-0.830576	-1.181795
H	1.812748	0.934765	-1.124453
C	1.180054	-0.002512	0.725885
H	1.366296	-0.895652	1.310231
H	1.358495	0.863730	1.352113
C	3.488688	0.000669	-0.240137
F	4.218233	0.032184	-1.367809
F	3.865286	1.053629	0.514472
F	3.837883	-1.113341	0.436114

[$F_5C_5N(CH(CH_3)(CF_3))]^+$ (C_1)

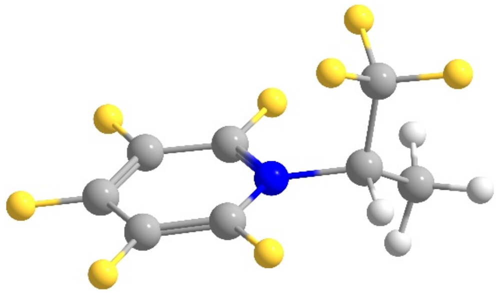

In the gas phase

C	0.595428	-1.201204	-0.371955
C	1.940395	-1.145190	-0.073962
C	2.527148	0.098706	0.153159
C	1.741299	1.246090	0.070137
C	0.399904	1.121095	-0.231962
F	-0.347329	2.181793	-0.303634
F	2.255940	2.434144	0.278300
F	3.792147	0.186740	0.438745
F	2.645706	-2.248483	-0.006616
F	0.022516	-2.351012	-0.588581
N	-0.175232	-0.086116	-0.460257
C	-1.656979	-0.246101	-0.755216
H	-1.752950	-1.267593	-1.104625
C	-2.195936	0.699256	-1.816966
H	-2.313076	1.716618	-1.462063
H	-1.554288	0.688786	-2.696169
H	-3.173104	0.325160	-2.115487
C	-2.406433	-0.190096	0.588758
F	-3.681451	-0.500213	0.385713
F	-1.870905	-1.074886	1.447156
F	-2.333169	1.021305	1.152050

With solvent model

C	0.610211	-1.208849	-0.321556
C	1.959985	-1.143776	-0.059872
C	2.546268	0.100575	0.140670
C	1.756324	1.241996	0.074861
C	0.411391	1.111348	-0.192122
F	-0.346290	2.167256	-0.242526
F	2.273424	2.435851	0.271022
F	3.822047	0.195132	0.395135
F	2.673504	-2.247365	0.002531
F	0.035080	-2.362327	-0.508355
N	-0.163440	-0.097500	-0.401216
C	-1.627027	-0.255423	-0.747871
H	-1.715748	-1.279732	-1.088552
C	-2.106084	0.671925	-1.853409
H	-2.224811	1.698267	-1.527266
H	-1.415112	0.630643	-2.691866
H	-3.069224	0.299050	-2.192812
C	-2.448663	-0.175709	0.546472
F	-3.719215	-0.489731	0.275937
F	-1.982.790	-1.044.831	1.458.772
F	-2.427.477	1.043.986	1.101.482

7 References

1 M. Hanack and J. Ullmann, *J. Org. Chem.*, 1989, **54**, 1432.

2 L. Fischer, M. H. Lee, I. Kim, A. Wiesner, K. F. Hoffmann and S. Riedel, *Angew. Chem. Int. Ed.*, 2024, e202407497.

3 K. Seppelt and D. Nothe, *Inorg. Chem.*, 1973, **12**, 2727.

4 D. Lentz and K. Seppelt, *Z. Anorg. Allg. Chem.*, 1983, **502**, 83.

5 OriginLab Corporation, OriginPro, Version 2022 (2022), Northampton, MA, USA.

6 G. M. Sheldrick, *Acta Cryst. A*, 2015, **71**, 3.

7 G. M. Sheldrick, *Acta Cryst. C*, 2015, **71**, 3.

8 O. V. Dolomanov, L. J. Bourhis, R. J. Gildea, J. A. K. Howard and H. Puschmann, *J. Appl. Cryst.*, 2009, **42**, 339.

9 H. Putz and K. Brandenburg, DIAMOND (4.6.4), Crystal Impact GbR, Bonn, 2020.

10 M. J. Frisch, G. W. Trucks, H. B. Schlegel, G. E. Scuseria, M. A. Robb, J. R. Cheeseman, G. Scalmani, V. Barone, G. A. Petersson, H. Nakatsuji, X. Li, M. Caricato, A. V. Marenich, J. Bloino, B. G. Janesko, R. Gomperts, B. Mennucci, H. P. Hratchian, J. V. Ortiz, A. F. Izmaylov, J. L. Sonnenberg, D. Williams-Young, F. Ding, F. Lipparini, F. Egidi, J. Goings, B. Peng, A. Petrone, T. Henderson, D. Ranasinghe, V. G. Zakrzewski, J. Gao, N. Rega, G. Zheng, W. Liang, M. Hada, M. Ehara, K. Toyota, R. Fukuda, J. Hasegawa, M. Ishida, T. Nakajima, Y. Honda, O. Kitao, H. Nakai, T. Vreven, K. Throssell, J. A. Montgomery, Jr., J. E. Peralta, F. Ogliaro, M. J. Bearpark, J. J. Heyd, E. N. Brothers, K. N. Kudin, V. N. Staroverov, T. A. Keith, R. Kobayashi, J. Normand, K. Raghavachari, A. P. Rendell, J. C. Burant, S. S. Iyengar, J. Tomasi, M. Cossi, J. M. Millam, M. Klene, C. Adamo, R. Cammi, J. W. Ochterski, R. L. Martin, K. Morokuma, O. Farkas, J. B. Foresman, and D. J. Fox, Gaussian 16 (Revision A.03), Gaussian, Inc., Wallingford CT, 2016.

11 a) S. H. Vosko, L. Wilk and M. Nusair, *Can. J. Phys.*, 1980, **58**, 1200; b) C. Lee, W. Yang and R. G. Parr, *Phys. Rev. B*, 1988, **37**, 785; c) A. D. Becke, *Phys. Rev. A*, 1988, **38**, 3098;

12 T. H. Dunning, *J. Chem. Phys.*, 1989, **90**, 1007.

13 S. Grimme, J. Antony, S. Ehrlich and H. Krieg, *J. Chem. Phys.*, 2010, **132**, 154104.

14 G. A. Zhurko, Chemcraft - graphical program for visualization of quantum chemistry computations. https://www.chemcraftprog.com (1.8, build 640), Ivanovo (Russia), 2005.
